# Development of multiplex digital PCR assays for the detection of PIK3CA mutations in the plasma of metastatic breast cancer patients

**DOI:** 10.1038/s41598-021-96644-6

**Published:** 2021-08-27

**Authors:** Julien Corné, Fanny Le Du, Véronique Quillien, Florence Godey, Lucie Robert, Héloïse Bourien, Angélique Brunot, Laurence Crouzet, Christophe Perrin, Claudia Lefeuvre-Plesse, Véronique Diéras, Thibault De la Motte Rouge

**Affiliations:** 1 Department of Biology, Centre Eugène Marquis, Unicancer, Rennes, France; 2 Department of Medical Oncology, Centre Eugène Marquis, Unicancer, Rennes, France; 3 INSERM U1242, University of Rennes, Rennes, France

**Keywords:** Cancer, Genetics, Molecular biology, Biomarkers, Oncology

## Abstract

With the approval of new therapies targeting the PI3K pathway, the detection of PIK3CA mutations has become a key factor in treatment management for HR+/HER2− metastatic breast cancer (MBC). We developed multiplex digital PCR (dPCR) assays to detect and quantify PIK3CA mutations. A first screening assay allows the detection of 21 mutations, with a drop-off system targeting the 542–546 hotspot mutations combined with the simultaneous detection of N345K, C420R, H1047L and H1047R mutations. In the case of a positive result, a sequential strategy based on other assays that we have developped allows for precise mutation identification. Clinical validity was determined by analyzing plasma circulating free DNA (cfDNA) from 213 HR+/HER2− MBC samples, as well as DNA extracted from 97 available matched tumors from 89 patients. Our assays have shown reliable specificity, accuracy and reproducibility, with limits of blank of three and four droplets for the screening assay. Sixty-eight patients (32%) had at least one PIK3CA mutation detectable in their plasma, and we obtained 83.1% agreement between the cfDNA analysis and the corresponding tumors. The high sensitivity and robustness of these new dPCR assays make them well-suited for rapid and cost-effective detection of PIK3CA mutations in the plasma of MBC patients.

## Introduction

The *PIK3CA* (phosphatidylinositol-4,5-bisphosphate 3-kinase catalytic subunit alpha) gene encoding the phosphatidylinositol-3-kinase (PI3K) catalytic subunit p110-alpha is commonly mutated in breast cancer. This often leads to hyperactivation of the PI3K/AKT/mTOR pathway, which plays a critical role in several cellular processes linked to oncogenesis, like migration, metabolism, cell growth and proliferation. Several drugs have been developed to target this pathway, among which three have been tested in large phase III clinical trials involving pretreated hormone receptor positive, human epidermal growth factor receptor 2 negative (HR+/HER2−) locally advanced or metastatic breast cancer (MBC) patients.

Pan-class I PI3K inhibitors have been tested in three trials: BELLE-2 (NCT01610284)^1^ and BELLE-3 (NCT01633060)^2^ for buparlisib; SANDPIPER^3^ (NCT02340221) for taselisib. In these trials, a significantly longer progression-free survival (PFS) with PI3K inhibitor compared to a placebo was observed; however, this benefit came at the cost of increased toxicity, and as a result these drugs are no longer in development.

Alpelisib is an isoform-specific PI3Kα inhibitor which has been tested in the SOLAR-1 trial (NCT02437318). Patients in this trial who had received prior endocrine therapy received either a combination of alpelisib/fulvestrant (a syntetic estrogen receptor antagonist) or placebo/fulvestrant. As for BELLE studies, tumor PIK3CA mutation status was found to be a predictive factor of response for PFS^4^. Final overall survival (OS) results have shown a 7.9 months numeric improvement for this group of patients^5^. The first results of the trial prompted FDA approval in May 2019 of alpelisib in combination with fulvestrant for postmenopausal women and men with HR+/HER2−, PIK3CA-mutated, advanced or metastatic breast cancer following progression while on or after treatment with an endocrine-based regimen. In 2020, the European Medicines Agency (EMA) in turn granted marketing authorization for alpelisib.

Detection of PIK3CA mutations is therefore becoming a crucial element for identifying patients most likely to benefit from alpelisib. Currently, there is no consensus concerning the best analytical method (liquid versus tissue) or the best type of biopsy (primary site versus metastasis). The most frequently used assays in clinical trials have been commercially available assays (Supplementary Table S1), with PIK3CA mutation status analyzed in tumors (mainly archival primary tumors) and circulating free DNA (cfDNA). Several arguments have called for the assessment of PIK3CA status in the metastatic setting, including arguments regarding (1) a risk of genomic evolution between the initial and metastatic tumor, (2) a risk of poor quality and/or quantity of DNA extracted from archival biopsies of formalin-fixed paraffin-embedded (FFPE) primary cancers stored for a long time. Since obtaining a metastatic tissue biopsy can be challenging for the clinician and/or uncomfortable for the patient, and given the emergence of very sensitive techniques, cfDNA assessment is an effective alternative to metastatic tumor analysis and has been recognized by the latest ESO-ESMO guidelines as an option for the selection of patients eligible for alpelisib^6^.

Digital PCR (dPCR) is a powerful technology for targeted mutation detection. It works by partitioning the sample into a large number of parallel PCR reactions (usually greater than 10,000) assuming that target DNA is distributed randomly into these partitions. After PCR amplification to end point, each partition is assigned as positive or negative, depending on the presence or absence of the amplified target sequence. The Poisson law is used to compute the average number of variant allele per partition, which leads to measure precisely its concentration with high sensitivity and reliability, without the need of calibration curves. This makes dPCR particularly well suited for liquid biopsy analysis.

A new strategy of PCR assay design called ‘drop-off’ enables the simultaneous detection of multiple mutations within genomic hotspots. A reference probe is designed to target an invariable region in the vicinity of the mutational hotspot. A drop-off probe is designed to target the wild-type sequence of the hotspot region. A double positive fluorescence signal indicates the presence of a wild-type allele. In case of a mutant allele, the binding of the drop-off probe is lost leading to a simple positive signal or sub-optimal, leading to a lower fluorescence amplitude, with droplets clearly distinguishable from those containing wild-type alleles. This type of assay has already been used in the field of cancer, for example to detect KRAS mutations in colon cancer and EFGR mutations in lung cancer^7^ or ESR1 mutation in breast cancer^8^.

Using the three-color Crystal dPCR™ platform (Stilla Technologies)^9^, we have designed a screening assay allowing the simultaneous detection of 21 PIK3CA mutations. This assay presents several advantages—including low cost and high sensitivity—which make it particularly well adapted for cfDNA assessment. We present here the analytical performances of this assay and our strategy to precisely identify the PIK3CA mutations in cases of a positive screening assay, as well as the results obtained from a large series of plasmas and tumors taken from HR+/HER2− MBC patients.

## Materials and methods

### Patients

A total of 213 HR+/HER2− MBC female patients treated in the Department of Medical Oncology of the Centre Eugène Marquis (CEM) in Rennes were included in this study (Supplementary Dataset). A blood sample was prospectively collected from each patient at the time of disease progression for cfDNA extraction. The results obtained for cfDNA were compared to those obtained for genomic DNA (gDNA) for 89 of these patients for which matched tumor samples were available. A total of 97 tumor samples (46 primary tumors and 51 metastatic) were assayed, with for each patient either one primary (38 patients), or one metastatic (43 patients), or one primary and one metastatic (8 patients) sample available. Tumor samples were either frozen samples (30) obtained from the processing of biological samples through the Centre de Ressources Biologiques (CRB)–Santé of Rennes (http://www.crbsante-rennes.com), or FFPE samples (67) that had been used for the histopathological diagnostic and stored in the Ouest Pathologie laboratory (Rennes). The research protocol was conducted under French legal guidelines and was approved by the medical ethics committee CREDO at the CEM. Written informed consent was obtained from all patients.

### Sample collection and processing

For cfDNA samples, 20 ml of blood were collected using two 10 ml K_2_EDTA blood collection tubes (BD Vacutainer®, Beckton, Dickinson) and processed within four hours of collection. Plasmas were obtained through double centrifugation at 1600*g* for 15 min and 4500*g* for 10 min, and were stored at −80 °C prior to cfDNA extraction. Frozen tumor tissue samples were stored in a freezer at −150 °C.

### Nucleic acid extractions and quantity assessments

CfDNA samples were extracted from 1.8 to 5 ml of plasma using the QIAamp Circulating Nucleic Acid kit (Qiagen), and were resuspended in a final volume of 50 μl of AVE buffer. Frozen tumor gDNA samples were extracted from two freshly-cut 10 µm sections using the QIAamp DNA Mini kit (Qiagen), and were resuspended in a final volume of 100 µl of AE buffer. FFPE tumor gDNA samples were extracted from two freshly-cut 10 µm sections using the QIAamp DNA FFPE Tissue kit (Qiagen), and were resuspended in a final volume of 50 µl of ATE buffer. The quantity of the extracted nucleic acids was assessed using the Qubit™ dsDNA HS Assay kit on a Qubit™ 3.0 Fluorometer (Thermo Fisher Scientific) (Supplementary Table S2).

### In silico* design and verifications of the PIK3CA assays*

All primers and hydrolysis probes (Supplementary Table S3) were designed using the software Primer3Plus with the sequences of the *PIK3CA* gene (NG_012113.2) for the studied mutations (Supplementary Table S4); they were then analyzed for gene specificity using Primer-BLAST. UNAfold and OligoAnalyzer webtools provided by Integrated DNA Technologies (IDT) were used for secondary structures, self-dimer and hetero-dimer predictions. OligoAnalyzer was also used to adjust the melting temperature (T_m_) of the hydrolysis probes via locked nucleic acids (LNA) substitutions in order to reach sufficient ΔT_m_ compared to the associated primer pairs, while also improving the specificity of detection. To further improve the specificity of H1047L and H1047R detections, a non-fluorescent blocker was designed with the corresponding wild-type (WT) sequence and a 3’-Phosphate modification. All oligonucleotides were synthesized by Eurogentec.

### Design of PIK3CA-mutated cfDNA-like positive controls

As positive controls, we designed gBlock® Gene Fragments (gBlock, IDT) of 166 bp in order to mimic the average cfDNA length (Supplementary Table S5). For the drop-off system, we selected the eight most frequent mutations occurring on codons 542–546, representing 98% of all the pathogenic mutations identified on this hotspot for breast carcinoma (BC) tumor samples in the catalogue of somatic mutations in cancer (COSMIC) database (Supplementary Figures S1 and S2).

### Other sources of DNA used during validation experiments

WT gDNA were extracted from the peripheral blood mononuclear cells (PBMC) of three healthy donors. For accuracy studies, we used the Quantitative Multiplex gDNA Reference Standard kit (Horizon Discovery).

### Digital PCR workflow

All dPCR experiments were performed with Sapphire Chips on the Naica™ system (Stilla Technologies). Each PCR was performed in a final volume of 25 µl, containing 5 µl of 5X PerfeCTa Multiplex qPCR ToughMix (Quanta BioSciences), 2.5 µl of 1 µM Fluorescein (VWR), 2.5 µl of homemade 10X PIK3CA assay (Supplementary Table S3) and 15 µl of input DNA. Highly concentrated samples were diluted in DNase/RNase Free UltraPure™ Distilled Water (Invitrogen) to reach a maximum theoretical concentration of 10,000 copies/PCR (33 ng/PCR) in order to limit background noises. Samples with low concentrations were assayed in two or three replicates to increase the sensitivity by investigating at least 10 ng/PCR. A negative H_2_O control and a positive control containing a mix of WT gDNA and mutated (MUT) gBlocks were included in every run. Each PCR program included an initial ‘partition’ step allowing for the formation of 15,000 to 30,000 droplets of 0.59 ± 0.03 nl, self-arranged into a crystal-like pattern, followed by PCR amplification cycles (Supplementary Table S6). The chips were imaged with the Naica™ Prism3 scanner using the Crystal Reader™ software v2.4.0.3 (Supplementary Table S7).

### Determination of the limits of blank (LOB_95%_) and the theoretical limits of detection (LOD_95%_)

Following the instructions provided by Stilla Technologies, we determined the limits of blank (LOB_95%_) and the theoretical limits of detection (LOD_95%_) for the PIK3CA assays by testing 30 replicates of WT-only samples (gDNA from healthy donors) with theoretical concentrations (based on the Qubit quantifications) of at least 10,000 copies/PCR, and calculating the means of the numbers of false positive droplets of each detection. The corrected means were then calculated using the following equation:

1 $${\mu }_{{{\text{corr}}}} = \upmu + 1.645 {\upsigma } \sqrt {\text{N}}$$,

 where μ is the mean, σ the standard deviation of false positive events and N the number of experiments performed. The LOB_95%_ were determined by fitting the *μ*_corr_ on Normal Law approximation and Chernoff’s inequality, and the LOD_95%_ were calculated using a similar approach as in Milbury et al.^10^ (Supplementary Table S8).

### Crystal dPCR™ data analysis

Analyses were performed using the Crystal Miner™ v2.4.0.3 software. We applied the quantification strategies defined for each assay, with specific polygon gates for droplets classification on the 2D dot plots. The results were ‘LOB-corrected’ in order to account for the potential presence of false positive droplets using the following equation:

2 $${\text{C}}_{{\text{(copies /}}\upmu{\rm l} {\text {\,of PCR Mix)}}} = \frac{{ - {\text{LN}}\left( {1{-}\frac{{{\text{k }}{-}{\text{ LOB}}_{{{{95\% }}}} }}{{{\text{N }}{-}{\text{ LOB}}_{{{{95\% }}}} }}} \right)}}{{{\text{V}}_{{{\text{Droplet}}}} }}$$,

 where ${\text{C}}_{{\text{(copies /}}\upmu{\rm l} {\text {of PCR Mix)}}}$ is the LOB-corrected concentration in copies/µl of PCR mix, ${\text{k}}$ the number of positive droplets, ${\text{N}}$ the number of total droplets and ${\text{V}}_{{{\text{Droplet}}}}$ the droplet volume (µl). The LOB-corrected concentrations were then converted into copies/ml of plasma using the following equation:

3 $${\text{C}}_{{\text{(copies/ml of plasma)}}} = \frac{{\left( {\frac{{{\text{ C}}_{{\text{(copies /}}\,\upmu{\rm l} {\text {\,of PCR Mix)}}} \times {\text{ V}}_{{\text{PCR mix}}} }}{{{\text{V}}_{{{\text{Input}}}} }}} \right) \times {\text{ V}}_{{{\text{Elution}}}} }}{{{\text{V}}_{{{\text{Plasma}}}} }}$$,

 with ${\text{V}}_{{\text{PCR mix}}} = 25\,\upmu{\rm l}$, ${\text{V}}_{{{\text{Elution}}}} = 50\,\upmu{\rm l}$,${\text{V}}_{{{\text{Input}}}}$ = volume (µl) of input DNA as mentioned above and ${\text{V}}_{{{\text{Plasma}}}}$ = volume (ml) of plasma. Only detections with a number of positive droplets higher or equal to the LOD_95%_ were considered positive. The mutant allelic frequencies (MAF) were determined using the following equation:

4 $${\text{MAF }}\left( \% \right) = \left( {\frac{{{\text{C}}_{{{\text{MUT}}}} }}{{{\text{C}}_{{{\text{WT}}}} + {\text{C}}_{{\text{MUT(s)}}} }}} \right) \times 100$$,

 where ${\text{C}}_{{{\text{MUT}}}}$ is the concentration of the considered mutation, ${\text{C}}_{{{\text{WT}}}}$ the concentration of WT DNA sequences and ${\text{C}}_{{\text{MUT(s)}}}$ the sum of the concentrations of all mutations detected.

### Statistical analysis

All statistical analyses were carried out using GraphPad Prism v8.0.0 (GraphPad Software).

## Results

**Figure 1:**
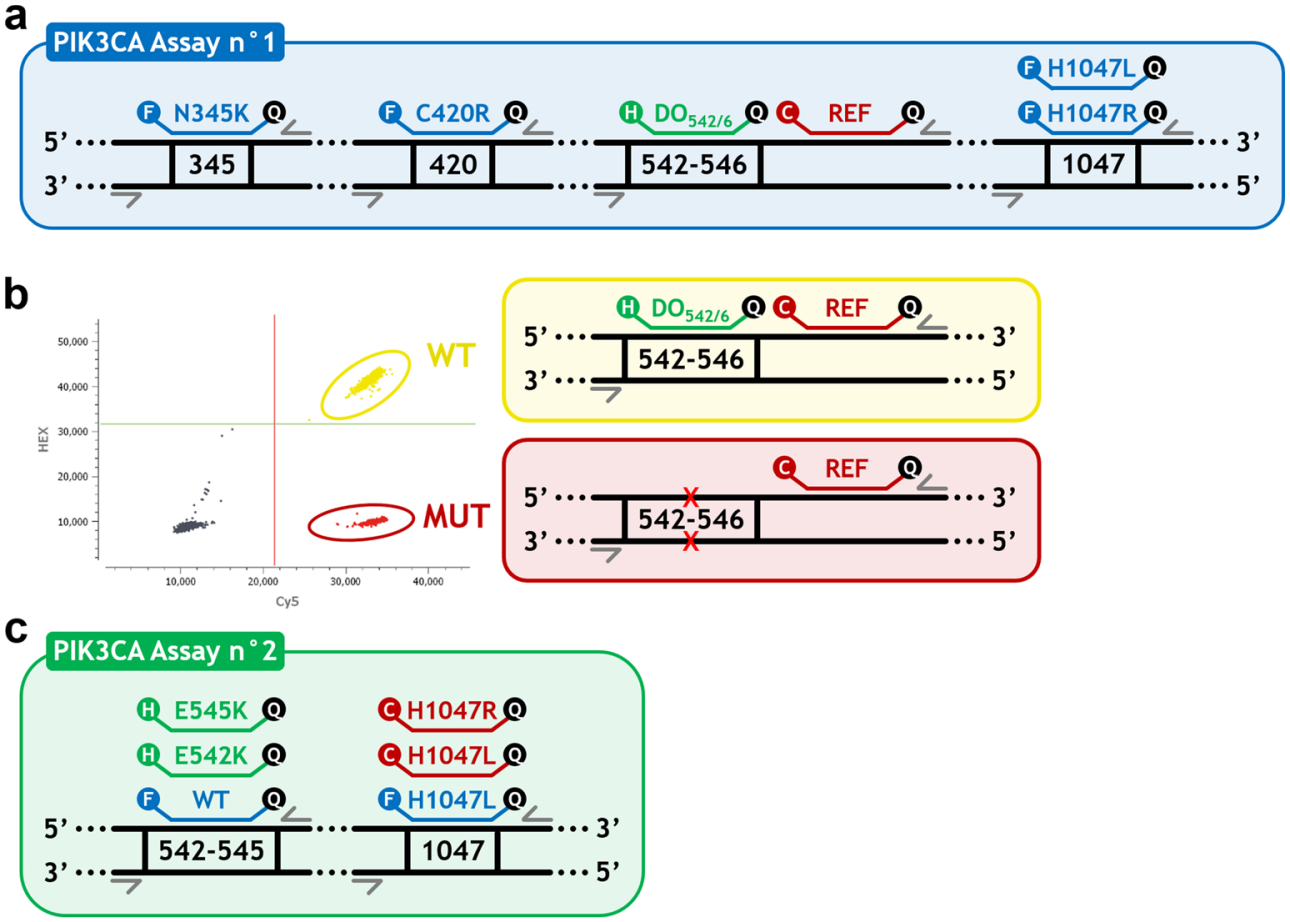
Design diagrams of the two multiplex assays for PIK3CA mutations detection and Drop-Off_542–546_ system principle. (**a**) Design diagram of the PIK3CA Assay n°1: four amplicons are simultaneously amplified using four couples of primers (grey arrows) to permit the detection of N345K, C420R, H1047L and H1047R mutations using FAM-labelled probes (blue color), as well as mutations on codons 542–546 using a HEX-labelled drop-off probe (green color) combined with a Cy5-labelled reference probe (red color). (**b**) Drop-Off_542–546_ system principle: WT sequences are detected by both the HEX-labelled drop-off probe and the Cy5-labelled reference probe, thus producing a cluster of HEX-Cy5 double-positive droplets (yellow-colored cluster on the 2D dot plot); whereas sequences bearing mutations (MUT, red crosses) on codons 542 to 546 have sub-optimal or even absent drop-off probe detection (red-colored cluster on the 2D dot plot). (**c**) Design diagram of the PIK3CA Assay n°2: two amplicons are simultaneously amplified using two couples of primers to permit the detection of wild-type (WT) sequences using a FAM-labelled probe, E542K and E545K mutations using HEX-labelled probes, H1047L mutation using FAM and Cy5-labelled probes and H1047R mutation using a Cy5-labelled probe (*C: Cy5; DO: drop-off; F: FAM; H: HEX; MUT: mutation; REF: reference*)*.*

Following the in silico design and verifications method described above, we designed two multiplex assays. The PIK3CA Assay n°1 (Fig. 1a), combines a drop-off system (Fig. 1b) for the detection of the 542–546 hotspot mutations (Drop-Off_542–546_) using a HEX-labelled drop-off probe covering the 542–546 hotspot and a Cy5-labelled reference probe located on the same amplicon, and simultaneous detection of N345K, C420R, H1047L and H1047R mutations, covering 90% of the pathogenic mutations identified for BC tumor samples in the COSMIC database (Supplementary Table S4). The PIK3CA Assay n°2 (Fig. 1c), includes the detection of the four most frequent mutations E542K, E545K, H1047L and H1047R.

### PIK3CA assays optimization

**Figure 2:**
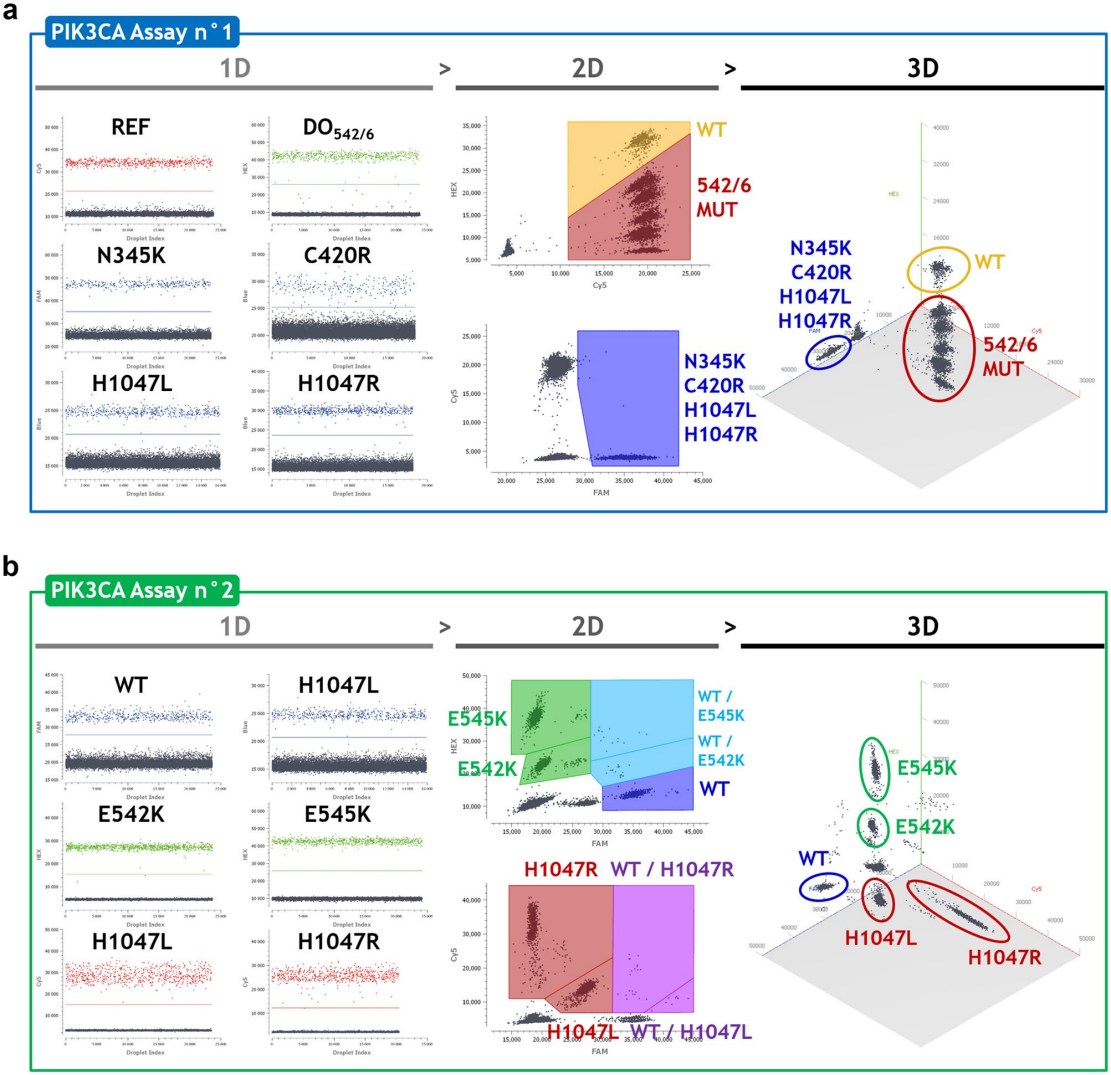
Optimization and quantification strategies for the PIK3CA assays. (**a**) PIK3CA Assay n°1: ‘1D’ left panel showing optimized fluorescence signals produced by each probe in a simplex reaction; ‘2D’ center panel showing the polygon gating quantification strategy defined using a mixture of WT gDNA and MUT gBlocks for N345K, C420R, E542K, E545A/G/K/Q, Q546K/P/R and H1047L/R mutations; ‘3D’ right panel showing the cluster positions according to their relative FAM-HEX-Cy5 fluorescence signals. (**b**) PIK3CA Assay n°2: ‘1D’ left panel showing optimized fluorescence signals produced by each probe in a simplex reaction; ‘2D’ center panel showing the polygon gating quantification strategy defined using a mixture of WT gDNA and MUT gBlocks for E542K, E545K and H1047L/R mutations; ‘3D’ right panel showing the cluster positions according to their relative FAM-HEX-Cy5 fluorescence signals.

We first checked the quality of the signals obtained in simplex reactions (Fig. 2, ‘1D’ left panels). The positive signals generated showed great separability from negative signals and very low amounts of ‘rain’ droplets. We then performed optimization experiments using mixtures of WT gDNA and MUT gBlocks to identify the optimal oligonucleotides concentrations (Supplementary Table S3), annealing/elongation temperatures and scanning parameters (Supplementary Table S6). We defined the quantification strategies using polygon gates for droplets classification on the 2D dot plots (Fig. 2, ‘2D’ center panels) with the help of the 3D visualization for cluster identification (Fig. 2, ‘3D’ right panels).

### PIK3CA assays validation

The LOB_95%_ and LOD_95%_ were determined as described above for one, two or three replicates (Supplementary Table S8). The LOB_95%_ for the PIK3CA Assay n°1 in one replicate were four droplets for the N345K-C420R-H1047L/R detection and three droplets for the 542/6_MUT detection. For the PIK3CA Assay n°2, we obtained ten, six, four and five droplets for the E542K, E545K, H1047L and H1047R detections, respectively. Samples with numbers of positive droplets between the LOB_95%_ and the LOD_95%_ were systematically investigated by performing two replicates, or three when needed, to increase the sensitivity.

**Figure 3:**
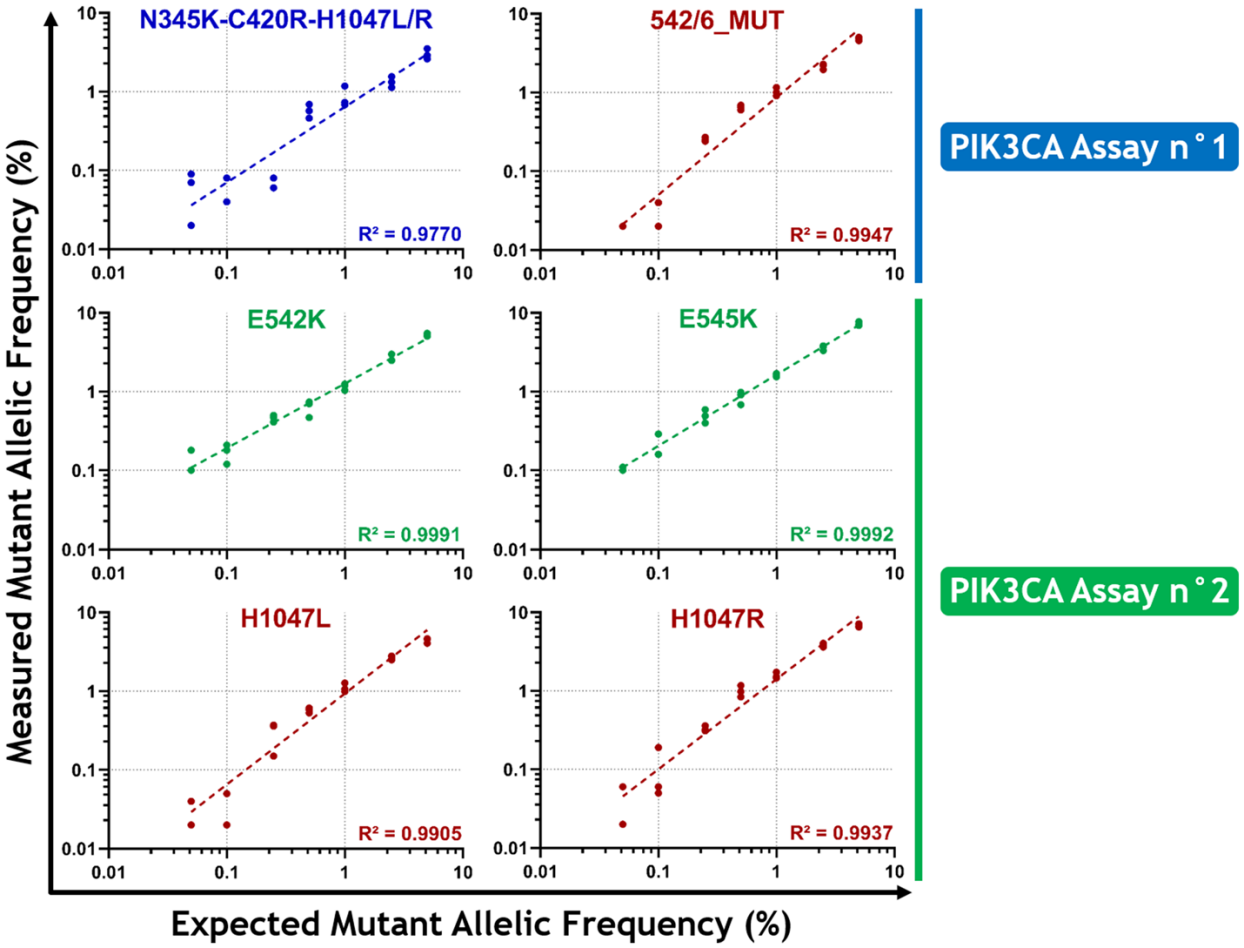
Evaluation of the sensitivity of the PIK3CA assays. DNA mixes were prepared through serial dilutions of MUT gBlocks in a constant WT gDNA background of 10,000 copies/PCR to reach theoretical MAF of 5%, 2.5%, 1%, 0.5%, 0.25%, 0.1% and 0.05%. The PIK3CA Assay n°1 mixes included the four MUT gBlocks of the N345K-C420R-H1047L/R detection and the eight most frequent mutations identified on codons 542–546 (*MAF: mutant allelic frequency*)*.*

For sensitivity analyses, DNA mixes were prepared using serial dilutions of MUT gBlocks in a constant WT gDNA background of 10,000 copies/PCR (Fig. 3). The DNA mixes were assayed in triplicate, except for the dilutions at MAF = 0.05% which were performed in quadruplicate. The coefficients of determination calculated for the linear regressions performed between expected and measured MAF of each detection ranged from R^2^ = 0.9770 to R^2^ = 0.9992. We considered positive any detection with at least two replicates that had equal or higher numbers of positive droplets than the corresponding LOD_95%_. Thus, we obtained sensitivities of 0.5% for N345K-C420R-H1047L/R and 0.25% for 542/6_MUT for the PIK3CA Assay n°1; and 0.1% for E542K and E545K, and 0.25% for H1047L and H1047R for the PIK3CA Assay n°2.

We evaluated the linearity of our assays over a dynamic range from 10,000 to 5 copies/PCR (Supplementary Figure S3). The coefficients of determination calculated for the linear regressions performed between expected and measured concentrations of each detection ranged from R^2^ = 0.9718 to R^2^ = 0.9995.

The coefficients of variation (CV) for repeatability ranged from 2.0 to 6.7% for 10,000 copies/PCR, from 2.6 to 4.2% for 5,000 copies/PCR and from 11.8 to 41.3% for 50 copies/PCR (Supplementary Table S9).

Inter-assay CV (reproducibility) were 8.9%, 8.7% and 9.8% for the WT, N345K-C420R-H1047L/R and 542/6_MUT detections, respectively, for the PIK3CA Assay n°1, and 9.3%, 8.1%, 8.1%, 9.7% and 8.2% for the WT, E542K, E545K, H1047L and H1047R detections, respectively, for the PIK3CA Assay n°2 (Supplementary Figure S4).

The accuracy performances were 90% for E545K and 81% for H1047R for the PIK3CA Assay n°1, and 96% for E545K and 93% for H1047R for the PIK3CA Assay n°2 (Supplementary Table S10).

The specificity of detection was characterized using a similar approach as in Milosevic et al.^11^, preparing DNA mixes with the corresponding MUT gBlocks at a fixed theoretical concentration of 50 copies/PCR and increasing amounts of WT gDNA from 0 to 10,000 copies/PCR. All mutation detections remained stable even in the presence of high concentrations of WT DNA (Supplementary Figure S5). We also performed cross-reactivity experiments to validate the specificity of detection for mutations involving competing probes, for which there was no impact on quantifications (data not shown).

Finally, we validated the analytical specificity (Supplementary Table S11) by first comparing the results obtained with the PIK3CA assays to those obtained with the commercial ‘ddPCR™ Mutation Assay: PIK3CA, Human, Homo sapiens’ from Bio-Rad for E542K, E545K and H1047R on the cfDNA samples of a small subset of 12 patients and obtained 100% concordance (7 mutated samples and 5 non-mutated). We also compared the PIK3CA assays results obtained on the cfDNA samples of a small subset of 4 patients to the the next-generation sequencing (NGS) results of the SAFIR02 study (NCT02299999) performed on tumor gDNA samples, and could confirm the presence of the same mutations for all of these patients, with the exception of a low frequency E545K mutation (MAF = 0.17%) found with the PIK3CA assays in plasma that could not be revealed by NGS in the tumor sample.

### Diagnostic strategy for PIK3CA mutations identification

**Figure 4:**
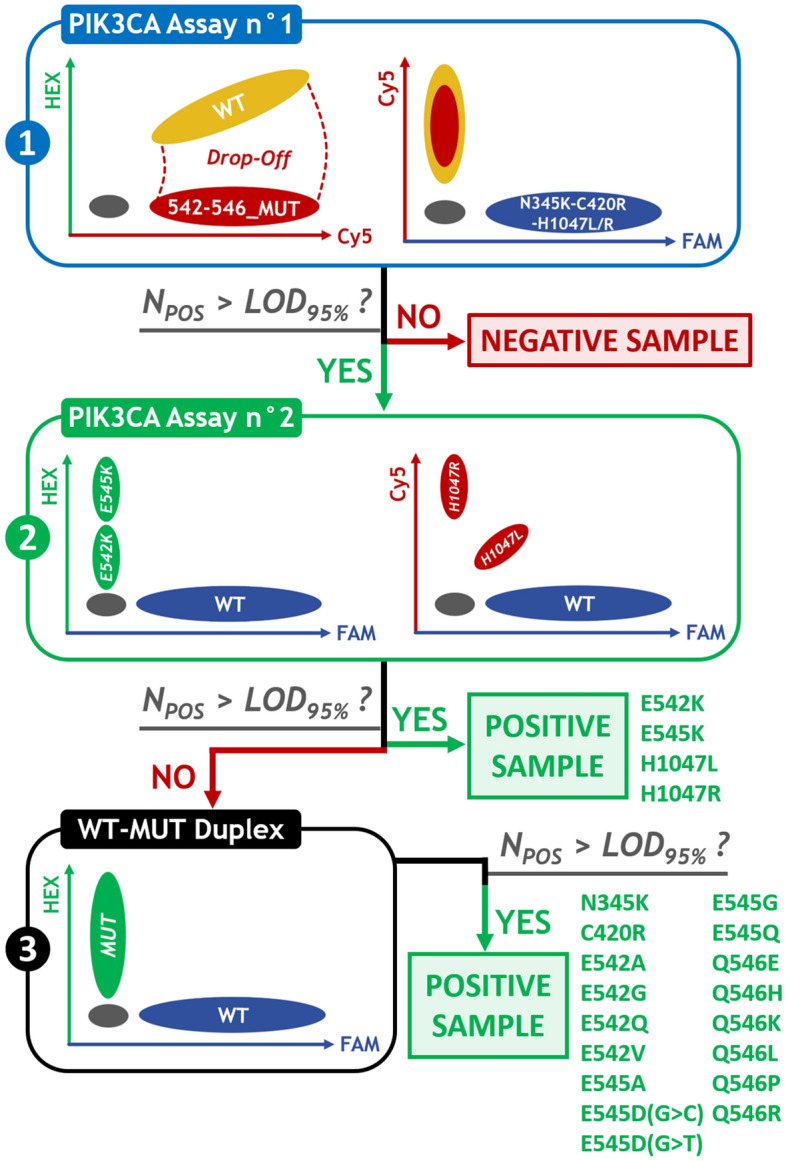
Diagnostic strategy for PIK3CA mutations identification. The PIK3CA Assay n°1, covering 90% of the PIK3CA mutations reported in the COSMIC database for breast carcinoma tumor samples, was performed as a first-line screening assay; followed, in case of a postive result, by the PIK3CA Assay n°2 focusing on the four most frequent PIK3CA mutations. Lastly, WT-MUT duplexes were performed for the identification of the less frequent mutations.

**Figure 5:**
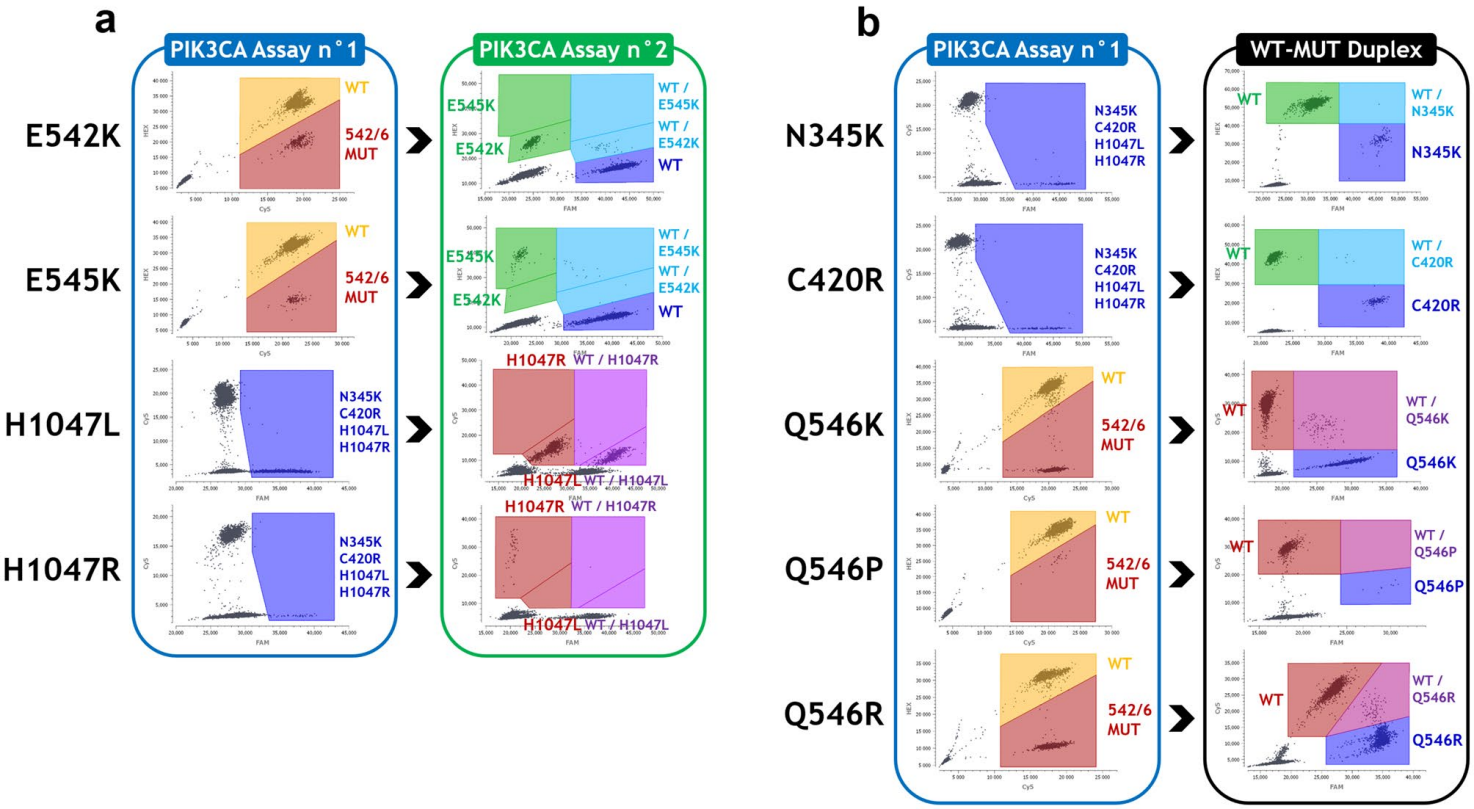
Examples of PIK3CA mutations identified on patient cfDNA samples with the PIK3CA assays and WT-MUT duplexes. (**a**) 2D dot plot results of the four most frequent PIK3CA mutations (E542K, E545K, H1047L and H1047R), first detected using the PIK3CA Assay n°1 with the respective MAF of 16.86%, 4.17%, 17.13% and 1.97%, and confirmed using the PIK3CA Assay n°2 with the respective MAF of 17.40%, 5.25%, 19.55% and 1.97%. (**b**) 2D dot plot results of the other mutations (N345K, C420R, Q546K, Q546P and Q546R), first detected using the PIK3CA Assay n°1 with the respective MAF of 4.00%, 3.83%, 35.02%, 1.12% and 39.62%, and confirmed using the WT-MUT duplexes with the respective MAF of 6.99%, 6.50%, 36.84%, 0.71% and 43.89%.

For the detection and identification of PIK3CA mutations, we developed a three-step diagnostic strategy (Fig. 4). We first performed the PIK3CA Assay n°1 as a screening assay. If no PIK3CA mutation was evidenced, samples were considered negative (Supplementary Figure S6). Otherwise, we performed the PIK3CA Assay n°2 (Fig. 5a). Finally, we performed individual WT-MUT Duplex assays as a third step for the less frequent PIK3CA mutations (Fig. 5b).

### PIK3CA assay results in patients

**Figure 6:**
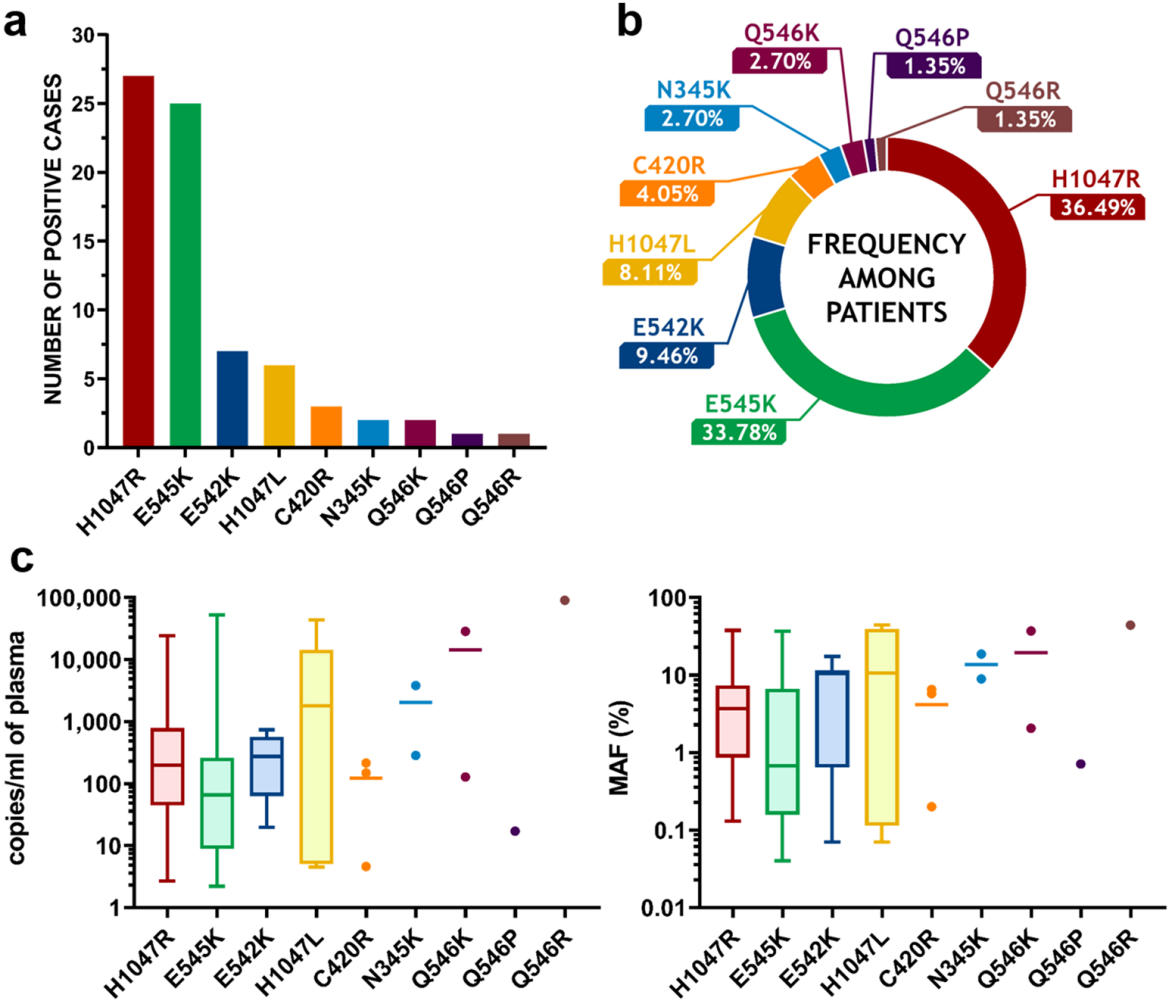
PIK3CA mutations detection in the plasma of 213 HR + /HER2− metastatic breast cancer patients. (**a**) Numbers of positive cases per mutation for the nine PIK3CA mutations found with the PIK3CA assays among the 213 HR + /HER2− metastatic breast cancer patients tested. (**b**) Relative frequencies of the nine PIK3CA mutations identified. (**c**) Distributions of the mutations in copies/ml of plasma and in MAF (%) (mutations with more than five instances are presented as box plots whereas mutations with less than five instances are presented as points, with median lines for more than two instances).

The median cfDNA concentration for the 213 plasma samples was 16 ng/ml of plasma (range 6–1120 ng/ml; mean: 50 ng/ml) (Supplementary Dataset). Sixty-eight patients (32%) harboured at least one mutation. A total of 74 mutations were found—six patients had two mutations per sample (Supplementary Figure S7). Among the 21 mutations potentially detected by our assays, nine were detected in our series of plasma samples with the following results: H1047R (27/74, 36.49%), E545K (25/74, 33.78%), E542K (7/74, 9.46%), H1047L (6/74, 8.11%), C420R (3/74, 4.05%), N345K (2/74, 2.70%), Q546K (2/74, 2.70%), Q546P (1/74, 1.35%) and Q546R (1/74, 1.35%) (Fig. 6a, b). Moreover, the relative frequencies obtained on our patients were rather consistent with the frequencies listed in the COSMIC database (Supplementary Table S12). The distribution of the quantifications obtained was quite broad for all mutations, both in copies/ml of plasma with the following results (median (min–max)): H1047R (196 (3–24,325)), E545K (66 (2–52,128)), E542K (273 (20–736)), H1047L (1782 (4–43,251)), C420R (148 (5–214)), N345K (2,048 (285–3812)), Q546K (14,244 (128–28,361)), Q546P (17) and Q546R (90,242); and in MAF (%) with the following values (median (min–max)): H1047R (3.70 (0.13–37.57)), E545K (0.67 (0.04–36.73)), E542K (10.42 (0.07–17.40)), H1047L (10.63 (0.07–44.19)), C420R (5.73 (0.20–6.50)), N345K (13.74 (8.88–18.59)), Q546K (19.54 (2.06–37.01)), Q546P (0.71) and Q546R (43,89) (Fig. 6c).

**Figure 7:**
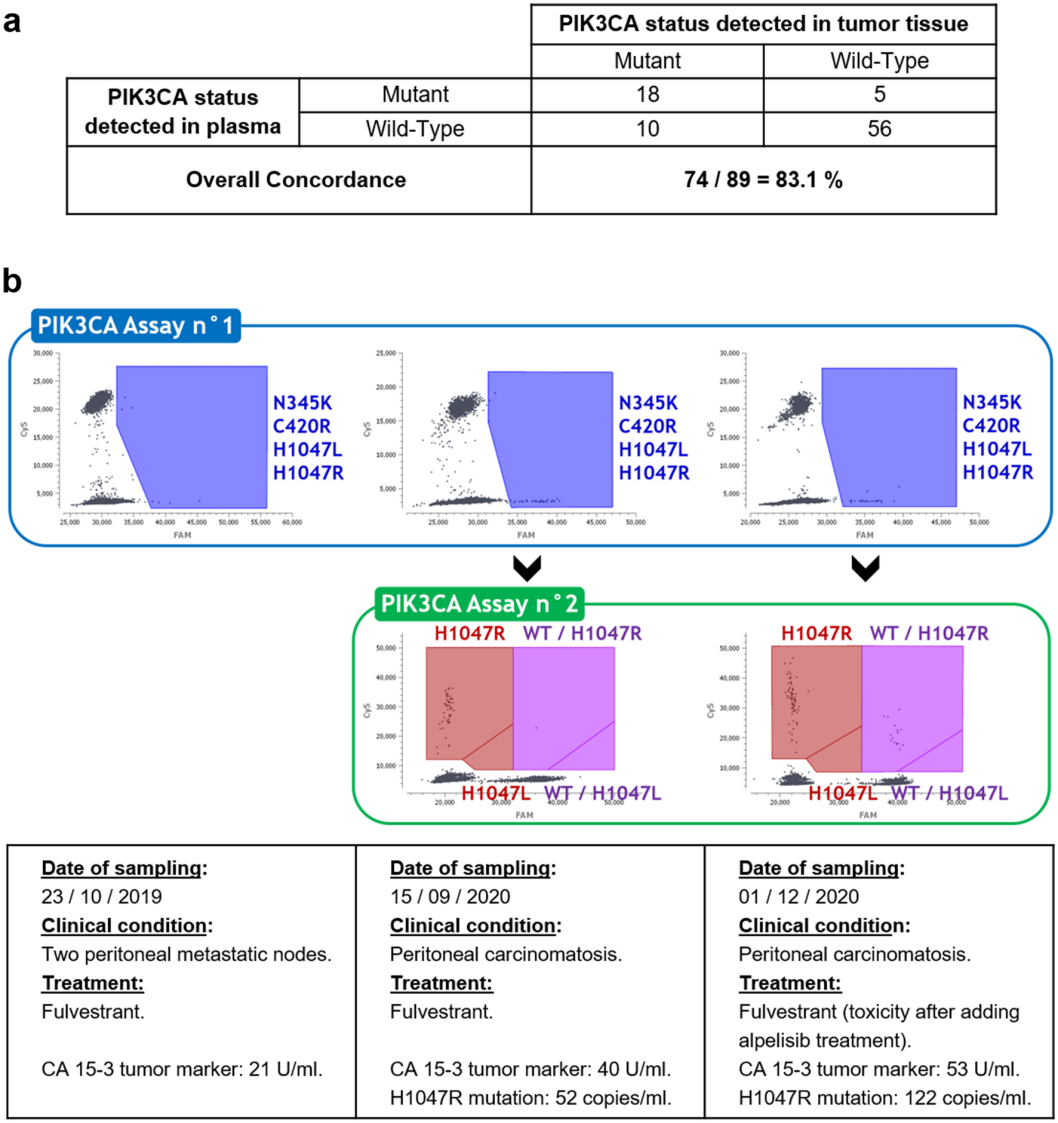
Clinical data on PIK3CA mutations detection and follow-up. (**a**) Comparison of PIK3CA status in tumor tissue and in plasma in a subset of 89 patients. (**b**) Example of a longitudinal follow-up in a patient, with the identification of an H1047R mutation that emerged during the course of the metastatic disease.

Matched tumors were available for 89 patients. We tested 46 initial tumors and 51 metastatic samples. For eight patients, both an initial and a metastatic sample were assayed (Supplementary Dataset). The median percentage of tumor cells for the samples was 70% (range 10–90%). The median time between tumor and plasma collections was 2.2 years (range 0–19.3 years). The concordance rate of PIK3CA mutation status between cfDNA and tumor tissue was 83.1% (Fig. 7a). The concordance rate was higher between breast tissue and plasma (87.0%, n = 46) compared to remote metastatic sites and plasma (77.8%, n = 36). For the 18 concordant positive patients, in each case the same mutation was found at the plasma and tumor levels. In five cases (5.6%), PIK3CA mutations were only found in plasma; in ten cases (11.2%), PIK3CA mutations were only found in the tumor tissue. We observed for some patients changes in results during the course of the metastatic disease; these were linked to changes in the tumor burden (Fig. 7b).

## Discussion

We have optimized highly sensitive, specific and robust multiplex dPCR assays which allow for rapid and cost-effective absolute quantification of the most frequent pathogenic PIK3CA mutations in breast cancer, with a coverage rate of 90%. Using those assays, we identified PIK3CA mutations in the plasma of 32% of HR+/HER2− female MBC patients, which is consistent with the results generally reported in the literature. In BELLE-2, BELLE-3 and SOLAR-1 trials, 32%, 34% and 29% of patients (respectively) had PIK3CA-mutated tumors^1,2,4^; 34% and 39% of patients in BELLE-2 and BELLE-3 had PIK3CA-mutated cfDNA^1,2^. In the SAFIR02 trial, 28% (104/364) of the metastatic HR+/HER2− tumors analyzed by NGS presented a PIK3CA mutation^12^. Although our assays do not identify rare PIK3CA mutations, it is not currently known whether the presence of such mutations is predictive of a response to alpelisib. In SOLAR-1 trials, tumors were analyzed by NGS; for the small group of patients (n = 31) with mutations only detectable by NGS, no predictive value was observed^13^. Of course, these results should be interpreted with caution given the small number of patients involved.

Liquid biopsy has many advantages, including the possibility of capturing tumor evolution with non-invasive procedures and better reflecting spatial heterogeneity, especially in cases of multiple metastatic sites. The question still remains as to whether it is necessary, in the event of a negative result on a liquid biopsy, to analyse the tumor in second intention. Such a procedure has been recommended by the FDA, following SOLAR-1 results. In this trial, tumors and plasmas were analyzed with the therascreen® PIK3CA kit, the sensitivity of which is much lower than that of dPCR. For example, the LOD for plasma specimens, defined as ‘the lowest amount of mutant DNA in a background of wild-type DNA at which a mutant sample will provide mutation positive results in 95% of the test results’, are 1.98% MAF for H1047R and 2.42% MAF for E545K for the therascreen® (Qiagen Handbook), when for these mutations we obtained sensitivities of 0.25% and 0.1% MAF, respectively.

Despite this high sensitivity, the concordance between paired plasmas and tumors in our study did not exceed 83.1%, which is comparable to what has been previously reported in the literature. Comparing metastatic lesions and temporally cfDNA samples pairs, 80% to 100% concordance rates have been reported^14–17^. In a study by Higgins et al.—the only study to use two different techniques for tissue and plasma—the concordance fell from 100 to 76% when the liquid and tissue samples were not collected simultaneously, and in more than half of the discordant cases mutations were only found in cfDNA^17^. Concordance rates of similar magnitude between matched circulating tumor DNA and tumor tissue were reported in the BELLE-2 (77%, n = 446 patients)^1^, BELLE-3 (83%, n = 256 patients)^2^ and SANDPIPER (80%, n = 508 patients)^3^ studies. In these clinical trials, the PIK3CA mutation status of tissue was mainly based on primary and not metastatic tumor samples.

Many explanations could account for these differences between cfDNA and tissue. (1) When the comparison is made between an initial tumor and plasma taken from the metastatic patient, this could be related to a change in PIK3CA mutation status upon disease recurrence. Indeed, some studies have reported differences between primary and paired asynchronous metastatic tumors. Examining 100 paired samples (with a majority of HR+/HER2− tumors), Dupont Jensen et al. reported that one-third of the patients displayed different results when looking for the presence of the three most common PIK3CA mutations, with predominantly a raise in mutations in the metastatic state^18^. However, in more recent studies comparing primary sites versus metastasis in HR+ patients, higher concordance rates have been reported: from 86 to 91%^19–22^. (2) In the case of multiple metastatic lesions, liquid biopsy can outperform single-lesion tumor biopsy in reflecting better tumor heterogeneity. This may explain why, in our study, the concordance between tissue and plasma was decreased when the comparison was made with a metastatic site. (3) One of the major advantages of liquid biopsy is the measurement of tumor dynamics. The longitudinal analysis carried out in our study illustrates it well for some patients, with either appearance of the mutation during the course of the disease or disappearance of the mutation under alpelisib treatment. This implies that the timing of the sampling is crucial.

Even if the results obtained by analyzing the plasma and the tumor are not completely super-imposable, it is the overall predictive value of the assay that should primarily be taken into account. Several studies of MBC with PI3K inhibitors or other mutation-directed therapies have now shown that cfDNA testing provides a predictive value of response to treatment at least as reliable as that provided with tumor analysis^1,2,5,23^. In the latest ESO-ESMO guidelines, cfDNA assessment is regarded as a good alternative to metastatic tumor analysis and is an option for the selection of patients eligible for alpelisib^6^. The high sensitivity, robustness and low cost (less than 1€ per test for reagents) of the multiplex dPCR assays we have developed make them suitable for the qualitative and quantitative clinical detection of PIK3CA mutations in plasma, and we can further speculate that the use of such a sensitive technique should make it possible to avoid reflex testing of the tumor in cases of a negative result on the plasma.

## Supplementary information

Dataset S1.

Supplementary Information.

## Data Availability

The data generated during the current study are available from the corresponding author on request.
