# Supplementary material for: Development of multiplex digital PCR assays for the detection of PIK3CA mutations in the plasma of metastatic breast cancer patients
Source: Sci Rep. 2021 Aug 27;11:17316. doi: 10.1038/s41598-021-96644-6 (PMC8397758; doi:10.1038/s41598-021-96644-6)
Supplement: Supplementary file 2 — Supplementary Information. [file 41598_2021_96644_MOESM2_ESM.docx]

**
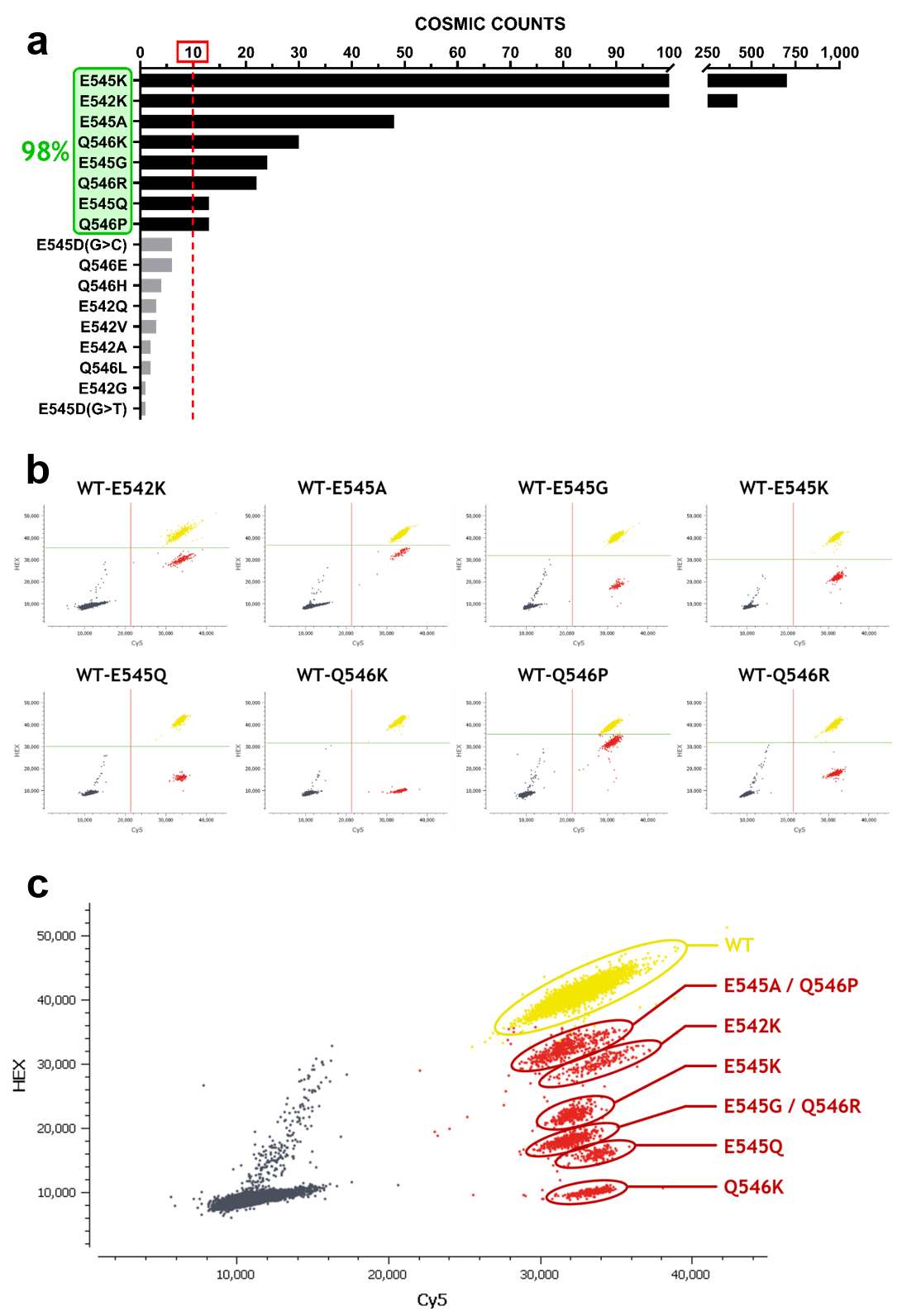
**

**Supplementary Figure S1. Mutated gBlocks selection for the 542/6_MUT detection of the PIK3CA Assay n°1 and relative positions of the MUT clusters obtained with the Drop-Off_542-546_ system.** (**a**) The 17 pathogenic PIK3CA mutations on codons 542 to 546 identified in the COSMIC database for breast carcinoma tumor samples were classified based on their relative COSMIC counts; a cut-off of ten counts was used to select the eight most frequent mutations representative of 98% of pathogenic PIK3CA mutations occurring on codons 542 to 546. (**b**) 2D dot plot results showing the individual cluster positions obtained for the eight selected MUT gBlocks with the Drop-Off_542-546_ system. (**c**) 2D dot plot results showing the superposition of all MUT cluster positions obtained for the eight selected MUT gBlocks with the Drop-Off_542-546_ system, creating a useful cartography for mutation identification based on cluster position.

**
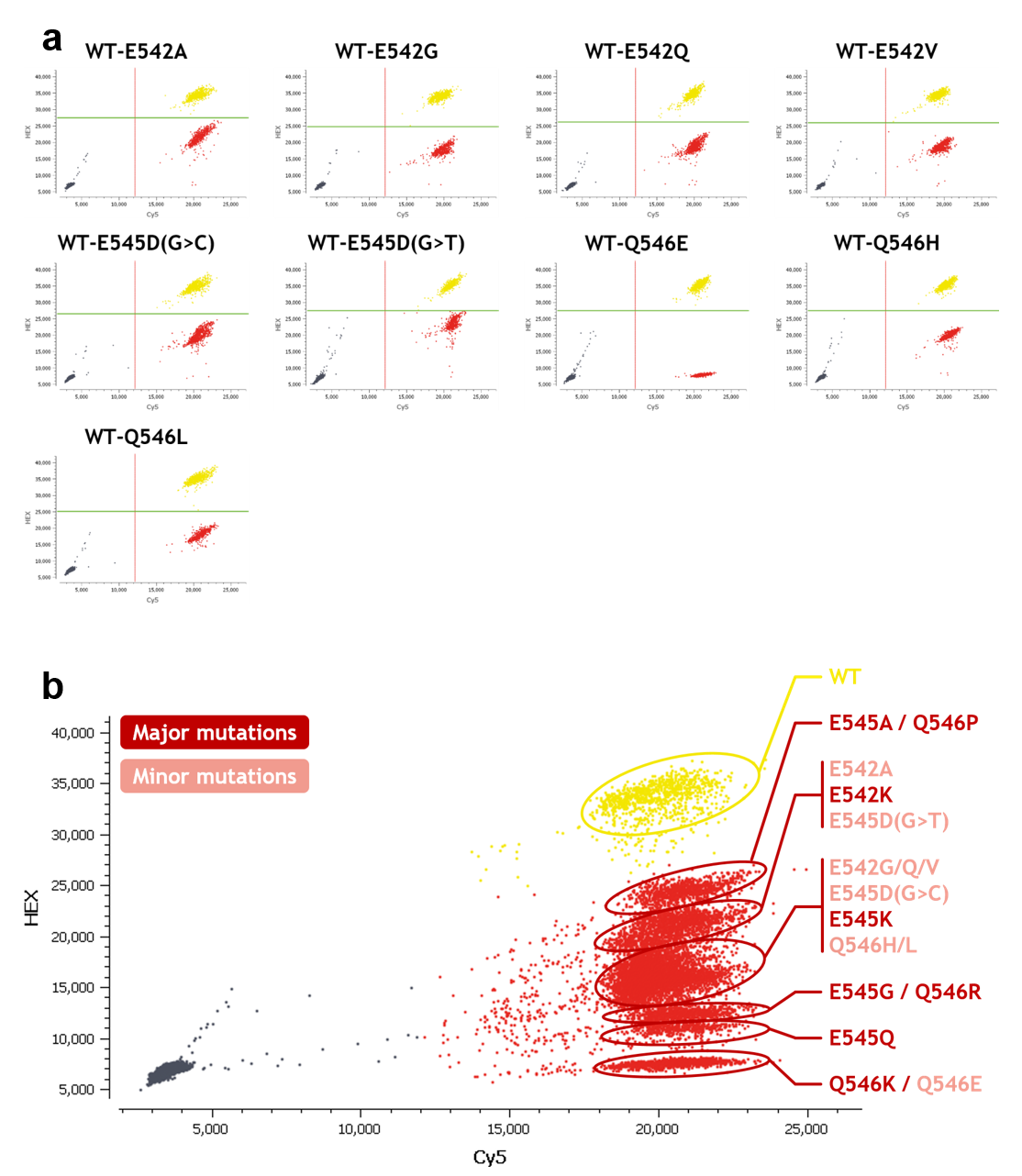
**

**Supplementary Figure S2. Complete cartography of the cluster positions obtained with the Drop-Off_542-546_ system for all pathogenic PIK3CA mutations.** (**a**) 2D dot plot results showing the individual cluster positions obtained for the nine other less frequent pathogenic PIK3CA mutations identified in the COSMIC database on codons 542 to 546. (**b**) 2D dot plot results showing the complete cartography of all cluster positions obtained with the Drop-Off_542-546_ system, including the eight most frequent pathogenic PIK3CA mutations (named ‘Major mutations’) as well as the nine less frequent pathogenic PIK3CA mutations (named ‘Minor mutations’).

**
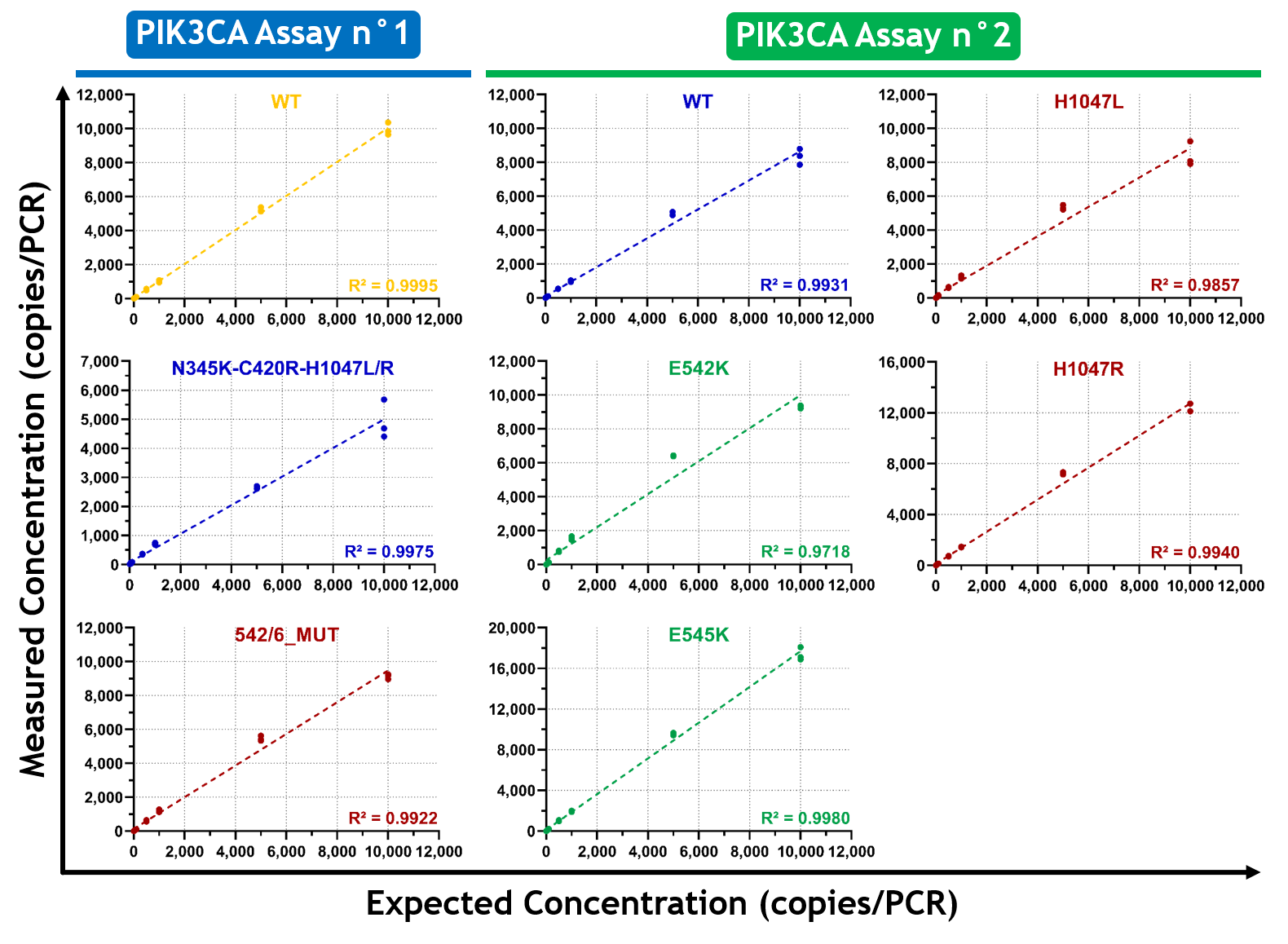
**

**Supplementary Figure S3. Evaluation of the linearity of the PIK3CA assays.** Eight DNA mixes were prepared for each multiplex assay through serial dilutions of MUT gBlocks and WT gDNA in order to obtain mixes with theoretical concentrations of 10,000, 5,000, 1,000, 500, 100, 50, 10 and 5 copies/PCR for each detection; these were assayed in triplicate. For the PIK3CA Assay n°1, a mix containing the four corresponding MUT gBlocks of the N345K-C420R-H1047L/R detection and the eight most frequent mutations identified on codons 542 to 546 (E542K, E545A/G/K/Q, Q546K/P/R) was prepared in order to reach the same theoretical concentrations considering the total amounts of MUT DNA per detection.

**
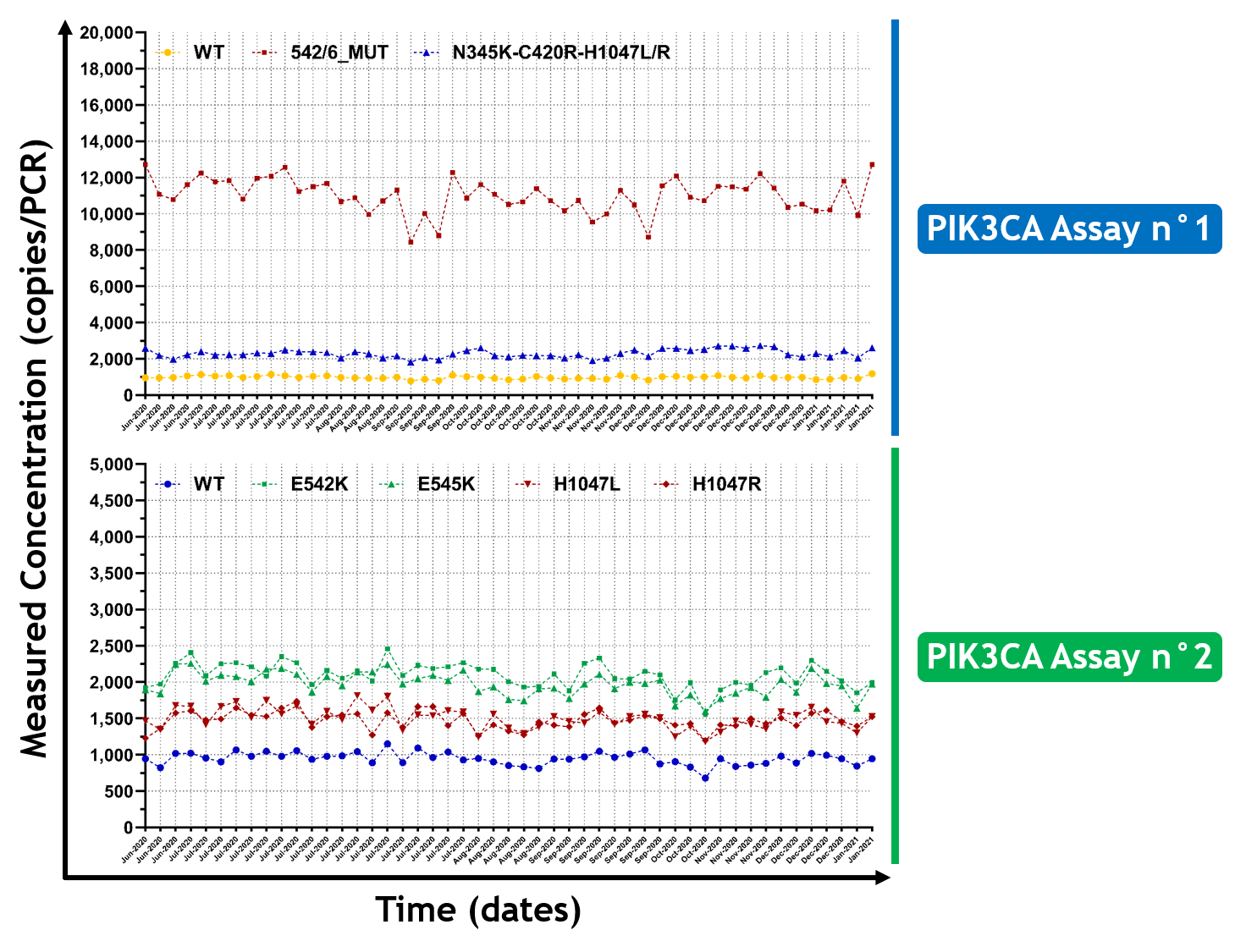
**

**Supplementary Figure S4. Evaluation of the reproducibility of the PIK3CA assays.** A DNA mix was prepared for each multiplex assay by assembling the corresponding MUT gBlocks and WT gDNA; these mixes were stored at -20°C in individual aliquots to serve as positive controls for each experiment. A total of 53 experiments were performed with the PIK3CA Assay n°1, and the coefficients of variation (CV) were 8.9%, 8.7% and 9.8% for the WT, N345K-C420R-H1047L/R and 542/6_MUT detections, respectively. A total of 49 experiments were performed with the PIK3CA Assay n°2 and the CV were 9.3%, 8.1%, 8.1%, 9.7% and 8.2% for the WT, E542K, E545K, H1047L and H1047R detections, respectively.

**
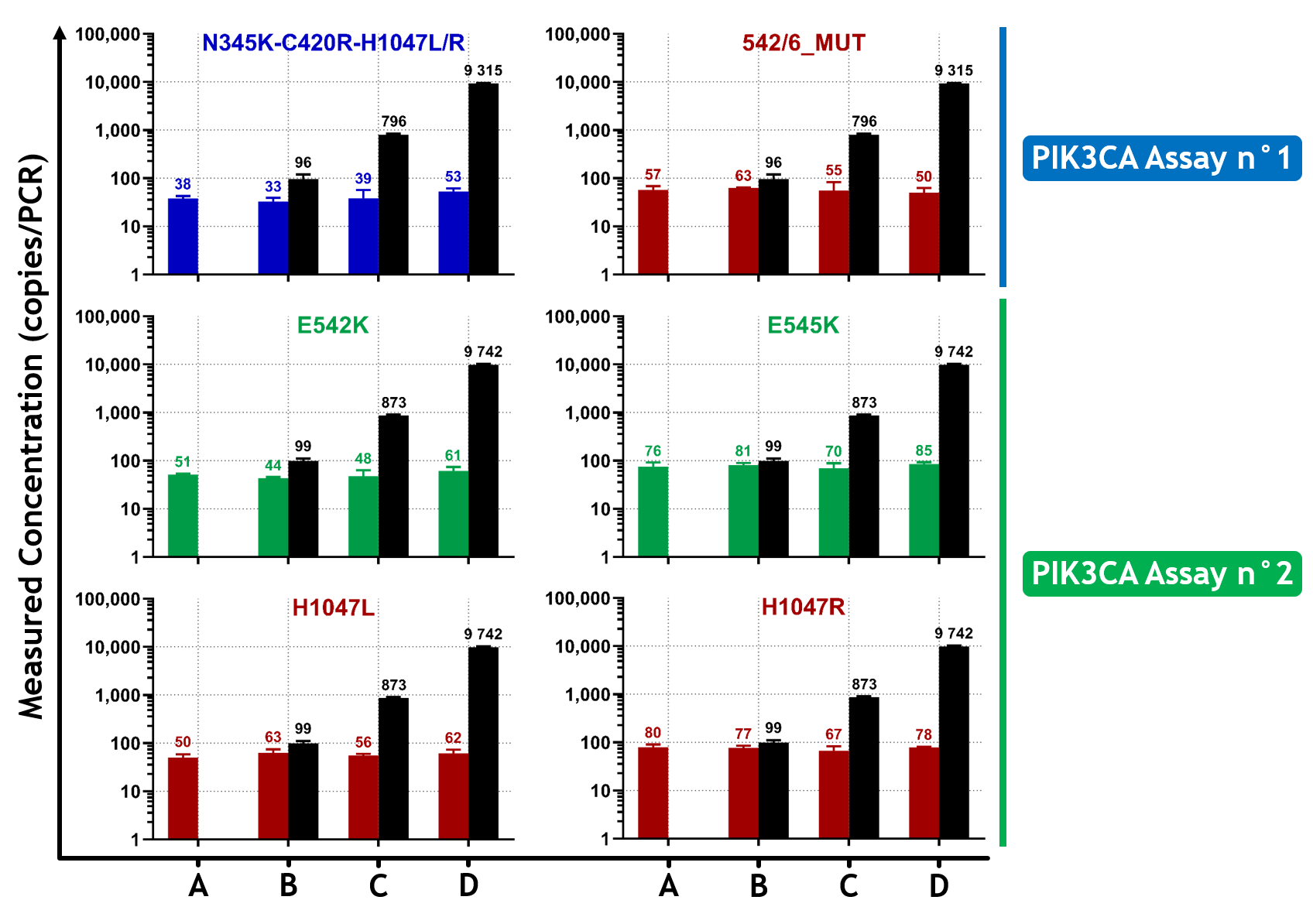
**

**Supplementary Figure S5.** **Evaluation of the specificity of the PIK3CA assays.** Four DNA mixes were prepared for each multiplex assay by assembling the corresponding MUT gBlocks with increasing amounts of WT gDNA to obtain the following theoretical concentrations of 0 (A), 100 (B), 1,000 (C) and 10,000 copies/PCR (D) for WT detections (black bars) with theoretical concentrations of 50 copies/PCR of the MUT gBlocks for all conditions (colored bars). The increasing amounts of WT gDNA had no impact on mutations detections.

**
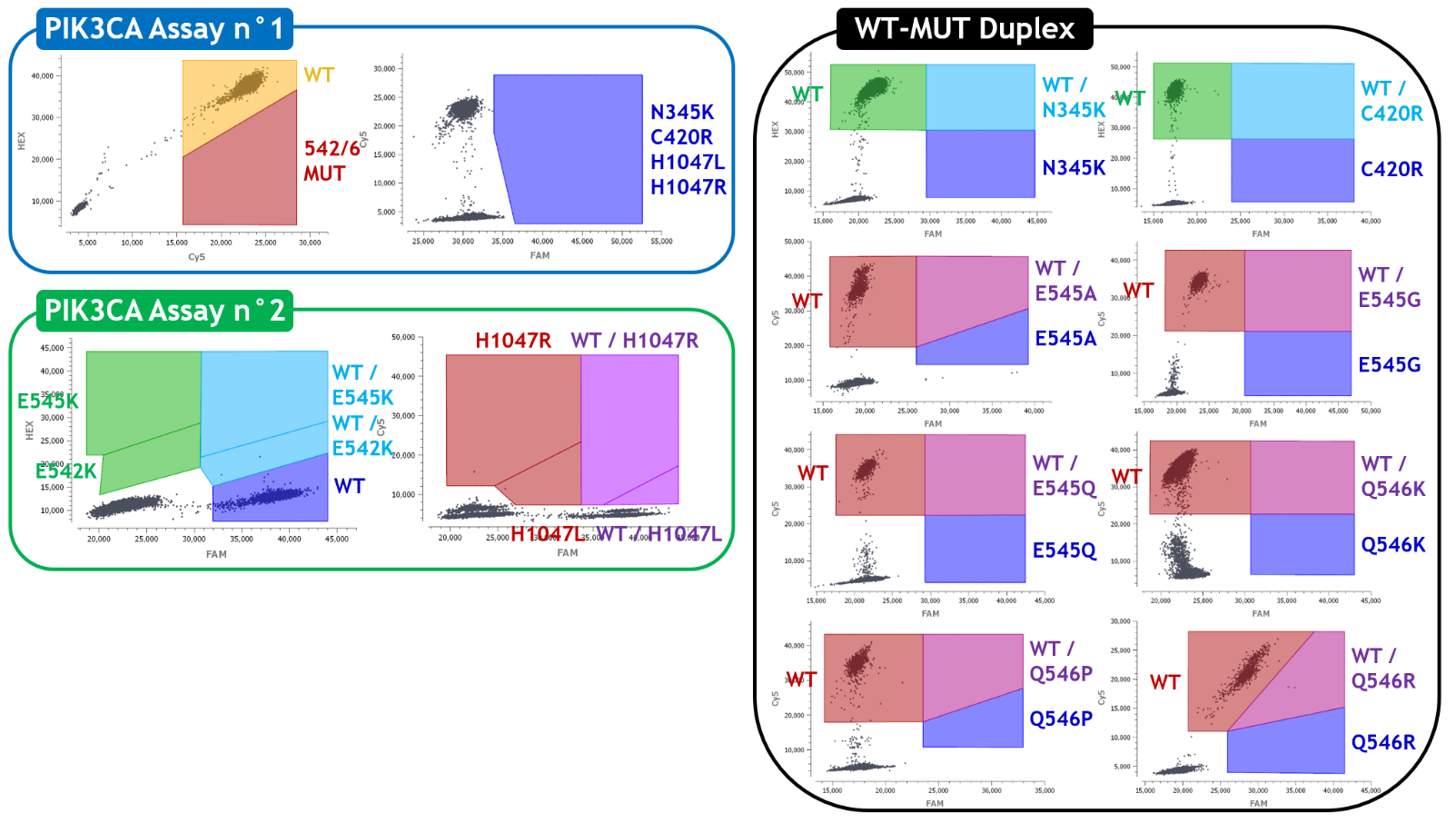
**

**Supplementary Figure S6. Examples of WT samples results obtained on cfDNA samples with the PIK3CA assays and WT-MUT duplexes.** 2D dot plot results showing examples of WT samples obtained with the PIK3CA Assay n°1, PIK3CA Assay n°2, as well as for the eight WT-MUT duplexes.

**
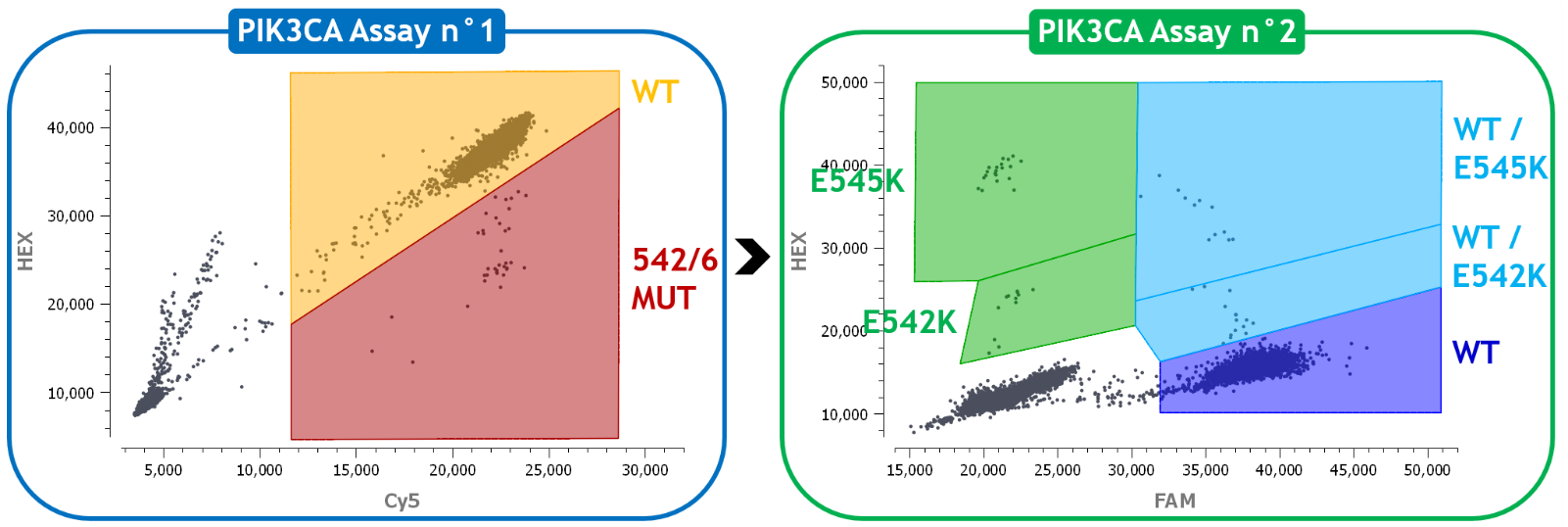
**

**Supplementary Figure S7. Example of the simultaneous detection of two mutations obtained on a cfDNA sample with the PIK3CA assays.** 2D dot plot results showing an example of a double-mutations (E542K, E545K) result obtained with the PIK3CA Assay n°1 (with the identification of two distinct clusters in the 542/6_MUT gate of quantification) and confirmed with the PIK3CA Assay n°2.

| **MUTATION** | | **PATHOGENIC SCORE (FATHMM*^a^* prediction)** | **MUTATION FREQUENCY FOUND IN COSMIC (%)** | **17**  **MUTATIONS DETECTED BY COBAS® TEST^b^** | **11**  **MUTATIONS DETECTED BY THERASCREEN® TEST^c^** | **8**  **MUTATIONS DETECTED BY INOSTICS BEAMING® TEST^d^ IN BELLE-3 STUDY** | **15**  **MUTATIONS DETECTED BY INOSTICS BEAMING® TEST^d^ IN BELLE-2 STUDY** | **21**  **MUTATIONS DETECTED BY OUR MULTIPLEX**  **dPCR ASSAY** |
| --- | --- | --- | --- | --- | --- | --- | --- | --- |
| **CDS** | **AA** |  |  |  |  |  |  |  |
| c.263G>A | R88Q | 0.96 | 0.16 | **YES** |  |  | **YES** |  |
| c.277C>T | R93W | n/a | 0.03 |  |  |  | **YES** |  |
| c.278G>A | R93Q | 0.99 | 0.03 |  |  |  | **YES** |  |
| c.331A>G | K111E | 0.98 | 0.19 |  |  |  | **YES** |  |
| c.333G>C | K111N | 0.96 | 0.05 |  |  |  | **YES** |  |
| c.353G>A | G118D | 1.00 | 0.36 |  |  |  | **YES** |  |
| c.1035T>G | N345K | 0.95 | 3.03 | **YES** |  |  |  | **YES** |
| c.1093G>A | E365K | 0.99 | 0.05 |  |  |  | **YES** |  |
| c.1258T>C | C420R | 0.98 | 1.45 | **YES** | **YES** |  | **YES** | **YES** |
| c.1624G>A | E542K | 0.97 | 11.48 | **YES** | **YES** | **YES** | **YES** | **YES** |
| c.1624G>C | E542Q | 0.98 | 0.08 |  |  |  |  | **YES** |
| c.1625A>T | E542V | 0.97 | 0.08 |  |  |  |  | **YES** |
| c.1624_1625delinsAG | E542R | n/a | 0.03 |  |  |  |  | **YES** |
| c.1625A>C | E542A | 0.97 | 0.05 |  |  |  |  | **YES** |
| c.1625A>G | E542G | 0.97 | 0.03 |  |  |  |  | **YES** |
| c.1633G>A | E545K | 0.97 | 19.14 | **YES** | **YES** | **YES** | **YES** | **YES** |
| c.1633G>C | E545Q | 0.98 | 0.36 |  |  |  |  | **YES** |
| c.1634A>C | E545A | 0.97 | 1.31 | **YES** | **YES** |  |  | **YES** |
| c.1634A>G | E545G | 0.97 | 0.66 | **YES** | **YES** | **YES** | **YES** | **YES** |
| c.1635G>C or G>T | E545D | 0.92 | 0.19 | **YES** | **YES** |  |  | **YES** |
| c.1636C>A | Q546K | 0.97 | 0.82 | **YES** |  | **YES** | **YES** | **YES** |
| c.1636G>C | Q546E | 0.97 | 0.16 | **YES** | **YES** |  |  | **YES** |
| c.1637A>G | Q546R | 0.97 | 0.6 | **YES** | **YES** |  |  | **YES** |
| c.1637A>T | Q546L | 0.97 | 0.05 | **YES** |  |  |  | **YES** |
| c.1637A>C | Q546P | 0.97 | 0.36 |  |  |  |  | **YES** |
| c.1638G>C or G>T | Q546H | 0.84 | 0.11 |  |  |  |  | **YES** |
| c.3129G>A or G>C or G>T | M1043I | 0.97 | 0.33 | **YES** |  | **YES** |  |  |
| c.3139C>T | H1047Y | 0.95 | 0.22 | **YES** | **YES** | **YES** | **YES** |  |
| c.3140A>T | H1047L | 0.96 | 5.44 | **YES** | **YES** | **YES** | **YES** | **YES** |
| c.3140A>G | H1047R | 0.96 | 42.65 | **YES** | **YES** | **YES** | **YES** | **YES** |
| c.3145G>C | G1049R | 0.97 | 0.52 | **YES** |  |  |  |  |

**Supplementary Table S1.** **Description of PIK3CA mutations detected in the most frequently used commercial assays and in our multiplex dPCR assay.**

*^a^ FATHMM: Functional Analysis Through Hidden Markov Model.*

*^b^ The cobas® PIK3CA Mutation Test (Roche Diagnostics), is a real-time polymerase chain reaction (PCR)-based assay.*

*^c^ The therascreen PIK3CA kit (Qiagen), is a real-time polymerase chain reaction (PCR)-based assay.*

*^d^ The BEAMING test (Sysmex Inostics), is a type of digital PCR that combines emulsion PCR and flow cytometry.*

*(AA: amino acid; CDS: coding sequence; n/a: information not available).*

| **ITEM TO CHECK** | **PROVIDED** | **COMMENT** |
| --- | --- | --- |
| **Column1** | **Y/N** | **Column2** |
| **1. SPECIMEN** |  |  |
| Detailed description of specimen type and numbers | **Y** | Materials and Methods |
| Sampling procedure (including time to storage) | **Y** | Materials and Methods |
| Sample aliquotation, storage conditions and duration | **Y** | Materials and Methods  Plasma samples were stored at -80°C in 5 ml cryotubes (Cryo.s^TM^, Greiner Bio-One International) for up to 26 months. |
| **2. NUCLEIC ACID EXTRACTION** |  |  |
| Description of extraction method including amount of sample processed | **Y** | Materials and Methods |
| Volume of solvent used to elute/resuspend extract | **Y** | Materials and Methods |
| Number of extraction replicates | **Y** | All nucleic acid extractions were performed in simplicates. |
| Extraction blanks included? | **N** | Extraction blanks were only performed during the validation experiments of the extraction procedures and were then not included in the following extraction series for economic considerations. |
| **3. NUCLEIC ACID ASSESSMENT AND STORAGE** |  |  |
| Method to evaluate quality of nucleic acids | **Y** | The quality of nucleic acids was assessed using the High Sensitivity DNA kit on a 2100 Bioanalyzer Instrument (Agilent Technologies) during the validation experiments of the extraction procedures and was then not realized in the following extraction series for economic considerations. |
| Method to evaluate quantity of nucleic acids (including molecular weight and calculations when using mass) | **Y** | Materials and Methods |
| Storage conditions: temperature, concentration, duration, buffer, aliquots | **Y** | For cfDNA samples: after quantification with the Qubit^TM^, each sample was divided in three aliquots of 16 µl stored at -80°C for up to 12 months, in the elution buffer.  For frozen and FFPE tumor gDNA samples: after quantification with the Qubit^TM^, each sample was stored at -80°C for up to 17 months, in the elution buffer. |
| Clear description of dilution steps used to prepare working DNA solution | **Y** | Materials and Methods |
| **4. NUCLEIC ACID MODIFICATION** | **NA** | NA |
| Template modification (digestion, sonication, pre-amplification, bisulphite etc.) | **NA** | NA |
| Details of repurification following modification if performed | **NA** | NA |
| **5. REVERSE TRANSCRIPTION** | **NA** | All templates measured with dPCR were DNA templates. |
| cDNA priming method and concentration | **NA** | NA |
| One or two step protocol (include reaction details for two step) | **NA** | NA |
| Amount of RNA added per reaction | **NA** | NA |
| Detailed reaction components and conditions | **NA** | NA |
| Estimated copies measured with and without addition of RT | **NA** | NA |
| Manufacturer of reagents used with catalogue and lot numbers | **NA** | NA |
| Storage of cDNA: temperature, concentration, duration, buffer and aliquots | **NA** | NA |
| **6. dPCR OLIGONUCLEOTIDES DESIGN AND TARGET INFORMATION** |  |  |
| Sequence accession number or official gene symbol | **Y** | Materials and Methods |
| Method (software) used for design and *in silico* verification | **Y** | Materials and Methods |
| Location of amplicon | **Y** | Materials and Methods; Supplementary Table S3 |
| Amplicon length | **Y** | Supplementary Table S3 |
| Primer and probe sequences (or amplicon context sequence) | **Y** | Supplementary Table S3 |
| Location and identity of any modifications | **Y** | Supplementary Table S3 |
| Manufacturer of oligonucleotides | **Y** | Materials and Methods |
| **7. dPCR PROTOCOL** |  |  |
| Manufacturer of dPCR instrument and instrument model | **Y** | Materials and Methods |
| Buffer/kit manufacturer with catalogue and lot number | **Y** | Materials and Methods (catalogue number: 95147-250; lot numbers: #028596, #017621, #66141243, #66145528, #66149494, #66157908, #66170964, Quanta BioSciences) |
| Primer and probe concentration | **Y** | Supplementary Table S3 |
| Pre-reaction volume and composition (incl. amount of template and if restriction enzyme added) | **Y** | Materials and Methods |
| Template treatment (initial heating or chemical denaturation) | **Y** | Materials and Methods |
| Polymerase identity and concentration, Mg++ and dNTP concentrations | **N** | Informations not available for commercial disclosure reasons. |
| Complete thermocycling parameters | **Y** | Supplementary Table S6 |
| **8. ASSAY VALIDATION** |  |  |
| Details of optimisation performed | **Y** | Materials and Methods; Results; Figure 2 |
| Analytical specificity (vs. related sequences) and limit of blank (LOB) | **Y** | Supplementary Table S8 |
| Analytical sensitivity/LoD and how this was evaluated | **Y** | Supplementary Table S8 |
| Testing for inhibitors (from biological matrix/extraction) | **Y** | Supplementary Figure S5 |
| **9. DATA ANALYSIS** |  |  |
| Description of dPCR experimental design | **Y** | Materials and Methods |
| Comprehensive details negative and positive of controls (whether applied for QC or for estimation of error) | **Y** | Materials and Methods |
| Partition classification method (thresholding) | **Y** | Materials and Methods; Results; Figure 2 |
| Examples of positive and negative experimental results (including fluorescence plots in Supplementary material) | **Y** | Results; Supplementary Figure S6 |
| Description of technical replication | **Y** | Supplementary Figure S4 |
| Repeatability (intra-experiment variation) | **Y** | Supplementary Table S9 |
| Reproducibility (inter-experiment/user/lab etc. variation) | **Y** | Supplementary Figure S4 |
| Number of partitions measured (average and standard deviation) | **Y** | The data generated during the current study are available from the corresponding author on request. |
| Partition volume | **Y** | 0.59 nl |
| Copies per partition (λ or equivalent) (average and standard deviation) | **Y** | The data generated during the current study are available from the corresponding author on request. |
| dPCR analysis program (source, version) | **Y** | Materials and Methods |
| Description of normalisation method | **NA** | NA |
| Statistical methods used for analysis | **Y** | Materials and Methods |
| Data transparency | **Y** | The data generated during the current study are available from the corresponding author on request. |

**Supplementary Table S2.** **Completed dMIQE2020 checklist.** *(NA: not applicable)*

| **PIK3CA Assay n°1** | | | | | | |
| --- | --- | --- | --- | --- | --- | --- |
| **OLIGO**  **NAME** | **OLIGO TYPE** | **5’-FLUOROPHORE** | **SEQUENCE**  **(5’ – 3’)** | **3’-MODIFICATION** | **AMPLICON LENGTH (bp)** | **FINAL CONCENTRATION (µM)** |
| 345_Fwd | Primer | NA | CCCTTTGGGTTATAAATAGTGC | NA | 90 | 0.8 |
| 345_Rev | Primer | NA | ACTTTACCTTATCAATGTCTCG | NA |  | 0.8 |
| 420_Fwd | Primer | NA | GAAATGTGTTTTATAATTTAGACTAGTGA | NA | 106 | 1 |
| 420_Rev | Primer | NA | GAGTGTCTGTGTAATCAAACAAG | NA |  | 1 |
| 542/6_Fwd | Primer | NA | AGCAATTTCTACACGAGATCCTC | NA | 86 | 0.75 |
| 542/6_Rev | Primer | NA | TCCATTTTAGCACTTACCTGTGAC | NA |  | 0.75 |
| 1047_Fwd | Primer | NA | GCAAGAGGCTTTGGAGTATT | NA | 73 | 0.75 |
| 1047_Rev | Primer | NA | ATCCATTTTTGTTGTCCAGC | NA |  | 0.75 |
| REF_Prb | Hydrolysis probe | Cy5 | ATTTT+CTA+T+G+GAG+T+CAC | BHQ-2 | NA | 0.15 |
| DO[542/6]_Prb | Hydrolysis probe | HEX | T+CTGAAATCA+CTGAGCAGG+AG | BHQ-1 | NA | 0.15 |
| N345K_Prb | Hydrolysis probe | FAM | C+CTA+CGTGAA+AGTAAATATT+CG | BHQ-1 | NA | 0.2 |
| C420R_Prb | Hydrolysis probe | FAM | A+GGAACAC+CGTCCAT+TG | BHQ-1 | NA | 0.6 |
| H1047L_Prb | Hydrolysis probe | FAM | T+GATGCA+C+TTC+ATGG+TG | BHQ-1 | NA | 0.5 |
| H1047R_Prb | Hydrolysis probe | FAM | T+GA+TGC+AC+GTCATGG+TG | BHQ-1 | NA | 0.2 |
| WT[1047]_Blk | Non-fluorescent blocker | NA | T+GATGCA+C+ATC+ATGG+TG | Phosphate | NA | 1.6 |
| **PIK3CA Assay n°2** | | | | | | |
| **OLIGO**  **NAME** | **OLIGO TYPE** | **5’-FLUOROPHORE** | **SEQUENCE**  **(5’ – 3’)** | **3’-MODIFICATION** | **AMPLICON LENGTH (bp)** | **FINAL CONCENTRATION (µM)** |
| 542/5_Fwd | Primer | NA | AGCAATTTCTACACGAGATCCTC | NA | 77 | 1 |
| 542/5_Rev | Primer | NA | GCACTTACCTGTGACTCCATAG | NA |  | 1 |
| 1047_Fwd | Primer | NA | GCAAGAGGCTTTGGAGTATT | NA | 73 | 1 |
| 1047_Rev | Primer | NA | ATCCATTTTTGTTGTCCAGC | NA |  | 1 |
| WT[542/5]_Prb | Hydrolysis probe | FAM | TC+T+GAAA+TCA+CT+GAGC | BHQ-1 | NA | 0.5 |
| E542K_Prb | Hydrolysis probe | HEX | T+C+T+AA+AAT+CA+CTGA+GC | BHQ-1 | NA | 0.45 |
| E545K_Prb | Hydrolysis probe | HEX | T+CTGAAA+T+C+A+CT+AA+GC | BHQ-1 | NA | 0.75 |
| H1047L_Prb | Hydrolysis probe | FAM | T+GATGCA+C+TTC+ATGG+TG | BHQ-1 | NA | 0.2 |
| H1047L_Prb(2) | Hydrolysis probe | Cy5 | TG+ATGCA+C+TT+CAT+GG | BHQ-2 | NA | 0.25 |
| H1047R_Prb(2) | Hydrolysis probe | Cy5 | TGATG+CA+C+GTC+ATGG | BHQ-2 | NA | 1.5 |
| WT[1047]_Blk | Non-fluorescent blocker | NA | T+GATGCA+C+ATC+ATGG+TG | Phosphate | NA | 2 |

***(Part 1 of 3)***

| **WT-N345K Duplex** | | | | | | |
| --- | --- | --- | --- | --- | --- | --- |
| **OLIGO**  **NAME** | **OLIGO TYPE** | **5’-FLUOROPHORE** | **SEQUENCE**  **(5’ – 3’)** | **3’-MODIFICATION** | **AMPLICON LENGTH (bp)** | **FINAL CONCENTRATION (µM)** |
| 345_Fwd | Primer | NA | CCCTTTGGGTTATAAATAGTGC | NA | 90 | 1 |
| 345_Rev | Primer | NA | ACTTTACCTTATCAATGTCTCG | NA |  | 1 |
| WT[345]_Prb | Hydrolysis probe | HEX | C+CTA+CGTGAA+TGTAAATATT+CG | BHQ-1 | NA | 0.25 |
| N345K_Prb | Hydrolysis probe | FAM | C+CTA+CGTGAA+AGTAAATATT+CG | BHQ-1 | NA | 0.25 |
| **WT-C420R Duplex** | | | | | | |
| **OLIGO**  **NAME** | **OLIGO TYPE** | **5’-FLUOROPHORE** | **SEQUENCE**  **(5’ – 3’)** | **3’-MODIFICATION** | **AMPLICON LENGTH (bp)** | **FINAL CONCENTRATION (µM)** |
| 420_Fwd | Primer | NA | GAAATGTGTTTTATAATTTAGACTAGTGA | NA | 106 | 1 |
| 420_Rev | Primer | NA | GAGTGTCTGTGTAATCAAACAAG | NA |  | 1 |
| WT[420]_Prb | Hydrolysis probe | HEX | A+GGAACA+C+TGTCCAT+TG | BHQ-1 | NA | 0.25 |
| C420R_Prb | Hydrolysis probe | FAM | A+GGAACAC+CGTCCAT+TG | BHQ-1 | NA | 0.25 |
| **WT-E545A Duplex** | | | | | | |
| **OLIGO**  **NAME** | **OLIGO TYPE** | **5’-FLUOROPHORE** | **SEQUENCE**  **(5’ – 3’)** | **3’-MODIFICATION** | **AMPLICON LENGTH (bp)** | **FINAL CONCENTRATION (µM)** |
| 542/6_Fwd | Primer | NA | AGCAATTTCTACACGAGATCCTC | NA | 86 | 1 |
| 542/6_Rev | Primer | NA | TCCATTTTAGCACTTACCTGTGAC | NA |  | 1 |
| WT[542/6]_Prb | Hydrolysis probe | Cy5 | CT+CT+GAAAT+CA+CT+GAGCA | BHQ-2 | NA | 0.25 |
| E545A_Prb | Hydrolysis probe | FAM | CT+CTGAA+AT+CA+CT+G+CG | BHQ-1 | NA | 0.25 |
| REF_Prb | Hydrolysis probe | Cy5 | ATTTT+CTA+T+G+GAG+T+CAC | BHQ-2 | NA | 0.25 |
| **WT-E545G Duplex** | | | | | | |
| **OLIGO**  **NAME** | **OLIGO TYPE** | **5’-FLUOROPHORE** | **SEQUENCE**  **(5’ – 3’)** | **3’-MODIFICATION** | **AMPLICON LENGTH (bp)** | **FINAL CONCENTRATION (µM)** |
| 542/6_Fwd | Primer | NA | AGCAATTTCTACACGAGATCCTC | NA | 86 | 1 |
| 542/6_Rev | Primer | NA | TCCATTTTAGCACTTACCTGTGAC | NA |  | 1 |
| WT[542/6]_Prb | Hydrolysis probe | Cy5 | CT+CT+GAAAT+CA+CT+GAGCA | BHQ-2 | NA | 0.25 |
| E545G_Prb | Hydrolysis probe | FAM | AATCA+CTG+GGCAGGA+GA | BHQ-1 | NA | 0.25 |

***(Part 2 of 3)***

| **WT-E545Q Duplex** | | | | | | |
| --- | --- | --- | --- | --- | --- | --- |
| **OLIGO**  **NAME** | **OLIGO TYPE** | **5’-FLUOROPHORE** | **SEQUENCE**  **(5’ – 3’)** | **3’-MODIFICATION** | **AMPLICON LENGTH (bp)** | **FINAL CONCENTRATION (µM)** |
| 542/6_Fwd | Primer | NA | AGCAATTTCTACACGAGATCCTC | NA | 86 | 1 |
| 542/6_Rev | Primer | NA | TCCATTTTAGCACTTACCTGTGAC | NA |  | 1 |
| WT[542/6]_Prb | Hydrolysis probe | Cy5 | CT+CT+GAAAT+CA+CT+GAGCA | BHQ-2 | NA | 0.25 |
| E545Q_Prb | Hydrolysis probe | FAM | AAAT+CA+CT+CA+GCAG+GAG | BHQ-1 | NA | 0.25 |
| **WT-Q546K Duplex** | | | | | | |
| **OLIGO**  **NAME** | **OLIGO TYPE** | **5’-FLUOROPHORE** | **SEQUENCE**  **(5’ – 3’)** | **3’-MODIFICATION** | **AMPLICON LENGTH (bp)** | **FINAL CONCENTRATION (µM)** |
| 542/6_Fwd | Primer | NA | AGCAATTTCTACACGAGATCCTC | NA | 86 | 1 |
| 542/6_Rev | Primer | NA | TCCATTTTAGCACTTACCTGTGAC | NA |  | 1 |
| WT[542/6]_Prb | Hydrolysis probe | Cy5 | CT+CT+GAAAT+CA+CT+GAGCA | BHQ-2 | NA | 0.25 |
| Q546K_Prb | Hydrolysis probe | FAM | T+CA+CTG+AGAAGG+A+GAAA | BHQ-1 | NA | 0.25 |
| **WT-Q546P Duplex** | | | | | | |
| **OLIGO**  **NAME** | **OLIGO TYPE** | **5’-FLUOROPHORE** | **SEQUENCE**  **(5’ – 3’)** | **3’-MODIFICATION** | **AMPLICON LENGTH (bp)** | **FINAL CONCENTRATION (µM)** |
| 542/6_Fwd | Primer | NA | AGCAATTTCTACACGAGATCCTC | NA | 86 | 1 |
| 542/6_Rev | Primer | NA | TCCATTTTAGCACTTACCTGTGAC | NA |  | 1 |
| WT[542/6]_Prb | Hydrolysis probe | Cy5 | CT+CT+GAAAT+CA+CT+GAGCA | BHQ-2 | NA | 0.25 |
| Q546P_Prb | Hydrolysis probe | FAM | TG+AAAT+CACT+GAG+C+CG | BHQ-1 | NA | 0.25 |
| **WT-Q546R Duplex** | | | | | | |
| **OLIGO**  **NAME** | **OLIGO TYPE** | **5’-FLUOROPHORE** | **SEQUENCE**  **(5’ – 3’)** | **3’-MODIFICATION** | **AMPLICON LENGTH (bp)** | **FINAL CONCENTRATION (µM)** |
| 542/6_Fwd | Primer | NA | AGCAATTTCTACACGAGATCCTC | NA | 86 | 1 |
| 542/6_Rev | Primer | NA | TCCATTTTAGCACTTACCTGTGAC | NA |  | 1 |
| WT[542/6]_Prb | Hydrolysis probe | Cy5 | CT+CT+GAAAT+CA+CT+GAGCA | BHQ-2 | NA | 0.25 |
| Q546R_Prb | Hydrolysis probe | FAM | CA+CTGAGC+GG+GAGAA+AG | BHQ-1 | NA | 0.25 |

***(Part 3 of 3)***

**Supplementary Table S3.** **Oligonucleotides composing the PIK3CA assays.** List of all the oligonucleotides (oligos) composing the PIK3CA assays (primers, hydrolysis probes and non-fluorescent blockers) with amplicon length provided for each primer pair, 5’- and 3’-modifications for probes and blockers and the final concentrations (µM) for each oligo.

*(NA: not applicable; +N: locked nucleic acid (LNA); Fwd: forward; Rev: reverse; Prb: probe; Blk: blocker; BHQ: black hole quencher).*

| **PIK3CA Assay n°1** | | | | | |
| --- | --- | --- | --- | --- | --- |
| **MUTATION** | | **COSMIC MUTATION ID** | **GENOMIC COORDINATES** | **COSMIC**  **COUNT** | **COSMIC COUNT**  **FREQUENCY (%)** |
| **CDS** | **AA** |  |  |  |  |
| c.3140A>G | p.H1047R | COSM775 | GRCh38, 3:179234297..179234297 | 1560 | 43.56 |
| c.1633G>A | p.E545K | COSM763 | GRCh38, 3:179218303..179218303 | 700 | 19.55 |
| c.1624G>A | p.E542K | COSM760 | GRCh38, 3:179218294..179218294 | 420 | 11.73 |
| c.3140A>T | p.H1047L | COSM776 | GRCh38, 3:179234297..179234297 | 199 | 5.56 |
| c.1035T>A | p.N345K | COSM754 | GRCh38, 3:179203765..179203765 | 111 | 3.1 |
| c.1258T>C | p.C420R | COSM267862 | GRCh38, 3:179210192..179210192 | 53 | 1.48 |
| c.1634A>C | p.E545A | COSM12458 | GRCh38, 3:179218304..179218304 | 48 | 1.34 |
| c.1636C>A | p.Q546K | COSM766 | GRCh38, 3:179218306..179218306 | 30 | 0.84 |
| c.1634A>G | p.E545G | COSM764 | GRCh38, 3:179218304..179218304 | 24 | 0.67 |
| c.1637A>G | p.Q546R | COSM12459 | GRCh38, 3:179218307..179218307 | 22 | 0.61 |
| c.1633G>C | p.E545Q | COSM27133 | GRCh38, 3:179218303..179218303 | 13 | 0.36 |
| c.1637A>C | p.Q546P | COSM1041496 | GRCh38, 3:179218307..179218307 | 13 | 0.36 |
| c.1635G>C | p.E545D | COSM27374 | GRCh38, 3:179218305..179218305 | 6 | 0.17 |
| c.1636C>G | p.Q546E | COSM6147 | GRCh38, 3:179218306..179218306 | 6 | 0.17 |
| c.1638G>T | p.Q546H | COSM24712 | GRCh38, 3:179218308..179218308 | 4 | 0.11 |
| c.1624G>C | p.E542Q | COSM17442 | GRCh38, 3:179218294..179218294 | 3 | 0.08 |
| c.1625A>T | p.E542V | COSM762 | GRCh38, 3:179218295..179218295 | 3 | 0.08 |
| c.1625A>C | p.E542A | COSM1041494 | GRCh38, 3:179218295..179218295 | 2 | 0.06 |
| c.1637A>T | p.Q546L | COSM25041 | GRCh38, 3:179218307..179218307 | 2 | 0.06 |
| c.1625A>G | p.E542G | COSM761 | GRCh38, 3:179218295..179218295 | 1 | 0.03 |
| c.1635G>T | p.E545D | COSM765 | GRCh38, 3:179218305..179218305 | 1 | 0.03 |
| **PIK3CA Assay n°2** | | | | | |
| **MUTATION** | | **COSMIC**  **MUTATION ID** | **GENOMIC COORDINATES** | **COSMIC**  **COUNT** | **COSMIC COUNT FREQUENCY (%)** |
| **CDS** | **AA** |  |  |  |  |
| c.3140A>G | p.H1047R | COSM775 | GRCh38, 3:179234297..179234297 | 1560 | 43.56 |
| c.1633G>A | p.E545K | COSM763 | GRCh38, 3:179218303..179218303 | 700 | 19.55 |
| c.1624G>A | p.E542K | COSM760 | GRCh38, 3:179218294..179218294 | 420 | 11.73 |
| c.3140A>T | p.H1047L | COSM776 | GRCh38, 3:179234297..179234297 | 199 | 5.56 |

**Supplementary Table S4.** **Mutations detected by the PIK3CA assays.** The exhaustive list of all the pathogenic PIK3CA mutations detected by the PIK3CA assays according to the information provided in the COSMIC database for breast carcinoma tumor samples, with their respective coding sequence (CDS) and amino acid (AA) characteristics, legacy mutation identifiers (ID), genomic coordinates on the genome reference consortium human build 38 (GRCh38), count and count frequency (%) calculated among all the pathogenic mutations. Thus, the PIK3CA Assay n°1 has a coverage rate of 90%.

| **gBlock NAME** | **SEQUENCE**  **(5’ – 3’)** | **LENGTH**  **(bp)** |
| --- | --- | --- |
| N345K_gBlock | tagtaatgcagacacttgcggtccatctcgCAAAATCCCTTTGGGTTATAAATAGTGCACTCAGAATAAAAATTCTTTGTGCAACCTACGTGAAAGTAAATATTCGAGACATTGATAAGGTAAAGTCAAATGCTGAagctgtcagcactactaacttgcggtcagt | 166 |
| C420R_gBlock | TTGGGGAAGAAAAGTGTTTTGAAATGTGTTTTATAATTTAGACTAGTGAATATTTTTCTTTGTTTTTTAAGGAACACCGTCCATTGGCATGGGGAAATATAAACTTGTTTGATTACACAGACACTCTAGTATCTGGAAAAATGGCTTTGAATCTTTGGCCAGTACC | 166 |
| E542A_gBlock | AGACTAGCTAGAGACAATGAATTAAGGGAAAATGACAAAGAACAGCTCAAAGCAATTTCTACACGAGATCCTCTCTCTGCAATCACTGAGCAGGAGAAAGATTTTCTATGGAGTCACAGGTAAGTGCTAAAATGGAGATTCTCTGTTTCTTTTTCTTTATTACAGA | 166 |
| E542G_gBlock | AGACTAGCTAGAGACAATGAATTAAGGGAAAATGACAAAGAACAGCTCAAAGCAATTTCTACACGAGATCCTCTCTCTGGAATCACTGAGCAGGAGAAAGATTTTCTATGGAGTCACAGGTAAGTGCTAAAATGGAGATTCTCTGTTTCTTTTTCTTTATTACAGA | 166 |
| E542K_gBlock | AGACTAGCTAGAGACAATGAATTAAGGGAAAATGACAAAGAACAGCTCAAAGCAATTTCTACACGAGATCCTCTCTCTAAAATCACTGAGCAGGAGAAAGATTTTCTATGGAGTCACAGGTAAGTGCTAAAATGGAGATTCTCTGTTTCTTTTTCTTTATTACAGA | 166 |
| E542Q_gBlock | AGACTAGCTAGAGACAATGAATTAAGGGAAAATGACAAAGAACAGCTCAAAGCAATTTCTACACGAGATCCTCTCTCTCAAATCACTGAGCAGGAGAAAGATTTTCTATGGAGTCACAGGTAAGTGCTAAAATGGAGATTCTCTGTTTCTTTTTCTTTATTACAGA | 166 |
| E542V_gBlock | AGACTAGCTAGAGACAATGAATTAAGGGAAAATGACAAAGAACAGCTCAAAGCAATTTCTACACGAGATCCTCTCTCTGTAATCACTGAGCAGGAGAAAGATTTTCTATGGAGTCACAGGTAAGTGCTAAAATGGAGATTCTCTGTTTCTTTTTCTTTATTACAGA | 166 |
| E545A_gBlock | AGACTAGCTAGAGACAATGAATTAAGGGAAAATGACAAAGAACAGCTCAAAGCAATTTCTACACGAGATCCTCTCTCTGAAATCACTGCGCAGGAGAAAGATTTTCTATGGAGTCACAGGTAAGTGCTAAAATGGAGATTCTCTGTTTCTTTTTCTTTATTACAGA | 166 |
| E545D(G>C)_gBlock | AGACTAGCTAGAGACAATGAATTAAGGGAAAATGACAAAGAACAGCTCAAAGCAATTTCTACACGAGATCCTCTCTCTGAAATCACTGACCAGGAGAAAGATTTTCTATGGAGTCACAGGTAAGTGCTAAAATGGAGATTCTCTGTTTCTTTTTCTTTATTACAGA | 166 |
| E545D(G>T)_gBlock | AGACTAGCTAGAGACAATGAATTAAGGGAAAATGACAAAGAACAGCTCAAAGCAATTTCTACACGAGATCCTCTCTCTGAAATCACTGATCAGGAGAAAGATTTTCTATGGAGTCACAGGTAAGTGCTAAAATGGAGATTCTCTGTTTCTTTTTCTTTATTACAGA | 166 |
| E545G_gBlock | AGACTAGCTAGAGACAATGAATTAAGGGAAAATGACAAAGAACAGCTCAAAGCAATTTCTACACGAGATCCTCTCTCTGAAATCACTGGGCAGGAGAAAGATTTTCTATGGAGTCACAGGTAAGTGCTAAAATGGAGATTCTCTGTTTCTTTTTCTTTATTACAGA | 166 |
| E545K_gBlock | AGACTAGCTAGAGACAATGAATTAAGGGAAAATGACAAAGAACAGCTCAAAGCAATTTCTACACGAGATCCTCTCTCTGAAATCACTAAGCAGGAGAAAGATTTTCTATGGAGTCACAGGTAAGTGCTAAAATGGAGATTCTCTGTTTCTTTTTCTTTATTACAGA | 166 |
| E545Q_gBlock | AGACTAGCTAGAGACAATGAATTAAGGGAAAATGACAAAGAACAGCTCAAAGCAATTTCTACACGAGATCCTCTCTCTGAAATCACTCAGCAGGAGAAAGATTTTCTATGGAGTCACAGGTAAGTGCTAAAATGGAGATTCTCTGTTTCTTTTTCTTTATTACAGA | 166 |
| Q546E_gBlock | AGACTAGCTAGAGACAATGAATTAAGGGAAAATGACAAAGAACAGCTCAAAGCAATTTCTACACGAGATCCTCTCTCTGAAATCACTGAGGAGGAGAAAGATTTTCTATGGAGTCACAGGTAAGTGCTAAAATGGAGATTCTCTGTTTCTTTTTCTTTATTACAGA | 166 |
| Q546H_gBlock | AGACTAGCTAGAGACAATGAATTAAGGGAAAATGACAAAGAACAGCTCAAAGCAATTTCTACACGAGATCCTCTCTCTGAAATCACTGAGCATGAGAAAGATTTTCTATGGAGTCACAGGTAAGTGCTAAAATGGAGATTCTCTGTTTCTTTTTCTTTATTACAGA | 166 |
| Q546K_gBlock | AGACTAGCTAGAGACAATGAATTAAGGGAAAATGACAAAGAACAGCTCAAAGCAATTTCTACACGAGATCCTCTCTCTGAAATCACTGAGAAGGAGAAAGATTTTCTATGGAGTCACAGGTAAGTGCTAAAATGGAGATTCTCTGTTTCTTTTTCTTTATTACAGA | 166 |
| Q546L_gBlock | AGACTAGCTAGAGACAATGAATTAAGGGAAAATGACAAAGAACAGCTCAAAGCAATTTCTACACGAGATCCTCTCTCTGAAATCACTGAGCTGGAGAAAGATTTTCTATGGAGTCACAGGTAAGTGCTAAAATGGAGATTCTCTGTTTCTTTTTCTTTATTACAGA | 166 |
| Q546P_gBlock | AGACTAGCTAGAGACAATGAATTAAGGGAAAATGACAAAGAACAGCTCAAAGCAATTTCTACACGAGATCCTCTCTCTGAAATCACTGAGCCGGAGAAAGATTTTCTATGGAGTCACAGGTAAGTGCTAAAATGGAGATTCTCTGTTTCTTTTTCTTTATTACAGA | 166 |
| Q546R_gBlock | AGACTAGCTAGAGACAATGAATTAAGGGAAAATGACAAAGAACAGCTCAAAGCAATTTCTACACGAGATCCTCTCTCTGAAATCACTGAGCGGGAGAAAGATTTTCTATGGAGTCACAGGTAAGTGCTAAAATGGAGATTCTCTGTTTCTTTTTCTTTATTACAGA | 166 |
| H1047L_gBlock | CATACATTCGAAAGACCCTAGCCTTAGATAAAACTGAGCAAGAGGCTTTGGAGTATTTCATGAAACAAATGAATGATGCACTTCATGGTGGCTGGACAACAAAAATGGATTGGATCTTCCACACAATTAAACAGCATGCATTGAACTGAAAAGATAACTGAGAAAA | 166 |
| H1047R_gBlock | CATACATTCGAAAGACCCTAGCCTTAGATAAAACTGAGCAAGAGGCTTTGGAGTATTTCATGAAACAAATGAATGATGCACGTCATGGTGGCTGGACAACAAAAATGGATTGGATCTTCCACACAATTAAACAGCATGCATTGAACTGAAAAGATAACTGAGAAAA | 166 |

**Supplementary Table S5.** **Mutated gBlocks used in this study.** List of the 21 mutated gBlocks used in this study and their nucleotide sequence. All gBlocks were designed to measure 166 bp in order to mimic the average cfDNA length. Mutated nucleotides are identified in red. The two sequences identified in lowercase and light blue for the N345K_gBlock correspond to synthesis adapters that were specifically required for the synthesis of this gBlock.

| **PIK3CA Assay n°1** | |
| --- | --- |
| **STEP** | **DETAILS** |
| **1** | Partition at 40°C, "Sapphire V1" |
| **2** | Temp. 95.0°C for 10' 0" Wait |
| **3** | Start Cycle, 45x |
| **4** | Temp. 95.0°C for 30" Wait |
| **5** | Temp. 64.0°C for 30" Wait |
| **6** | Close Cycle |
| **7** | Release P, "Sapphire V1" |
| **PIK3CA Assay n°2** | |
| **STEP** | **DETAILS** |
| **1** | Partition at 40°C, "Sapphire V1" |
| **2** | Temp. 95.0°C for 10' 0" Wait |
| **3** | Start Cycle, 45x |
| **4** | Temp. 95.0°C for 30" Wait |
| **5** | Temp. 60.0°C for 30" Wait |
| **6** | Close Cycle |
| **7** | Release P, "Sapphire V1" |

**Supplementary Table S6. PCR programs used for the PIK3CA assays.** Detailed steps of the PCR programs used for the PIK3CA assays on the Naica^TM^ Geode. The crystals of droplets were first generated during the ‘partition’ step (STEP 1) by an increase of pressure up to +1,000 mbar at a fixed temperature of 40°C, followed by the PCR amplification steps (STEP 2 to 6) and the final release step (STEP 7) to return to ambient temperature and pressure conditions. The PIK3CA Assay n°1 PCR program was also used for the WT-E545A, WT-E545G, WT-E545Q, WT-Q546K, WT-Q546P and WT-Q546R duplexes, and the PIK3CA Assay n°2 PCR program was used for the WT-N345K and WT-C420R duplexes.

| **PIK3CA Assay n°1** | |
| --- | --- |
| **PARAMETER** | **VALUE** |
| **FOCUS** | 0.74 mm |
| **FAM** | 90 ms |
| **HEX** | 250 ms |
| **Cy5** | 50 ms |
| **PIK3CA Assay n°2** | |
| **PARAMETER** | **VALUE** |
| **FOCUS** | 0.74 mm |
| **FAM** | 80 ms |
| **HEX** | 90 ms |
| **Cy5** | 30 ms |

**Supplementary Table S7. Scanning parameters applied for the PIK3CA assays.** Detailed values of the different parameters applied during the scanning of the Sapphire chips on the Naica^TM^ Prism3 for the PIK3CA assays. The scanning parameters applied for the WT-MUT duplexes were the same as for the PIK3CA Assay n°1, except for the WT-E545A Duplex which differed in a Cy5 excitation duration of 30 ms rather than 50 ms.

| **PIK3CA Assay n°1** | | | | | | | |
| --- | --- | --- | --- | --- | --- | --- | --- |
| **MUTATION DETECTION** | **NUMBER OF REPLICATES** | **LOB_95%_** | **LOD_95%_** | | | | |
|  |  | **droplets** | **droplets** | **copies/µl of PCR Mix** | **copies/PCR** | **copies/ml of plasma** | **MAF (%)** |
| **N345K-C420R-H1047L/R** | 1 | 4 | 9 | 0.789 | 19.7 | 13.2 | 0.197 |
|  | 2 | 6 | 12 | 0.514 | 25.7 | 8.6 | 0.129 |
|  | 3 | 8 | 15 | 0.419 | 31.4 | 7.0 | 0.105 |
| **542/6_MUT** | 1 | 3 | 8 | 0.665 | 16.6 | 11.1 | 0.166 |
|  | 2 | 5 | 11 | 0.455 | 22.8 | 7.6 | 0.114 |
|  | 3 | 6 | 12 | 0.342 | 25.7 | 5.7 | 0.086 |
| **PIK3CA Assay n°2** | | | | | | | |
| **MUTATION DETECTION** | **NUMBER OF REPLICATES** | **LOB_95%_** | **LOD_95%_** | | | | |
|  |  | **droplets** | **droplets** | **copies/µl of PCR Mix** | **copies/PCR** | **copies/ml of plasma** | **MAF (%)** |
| **E542K** | 1 | 10 | 17 | 1.310 | 32.8 | 21.8 | 0.328 |
|  | 2 | 17 | 26 | 0.989 | 49.5 | 16.5 | 0.247 |
|  | 3 | 23 | 33 | 0.845 | 63.4 | 14.1 | 0.211 |
| **E545K** | 1 | 6 | 12 | 0.909 | 22.7 | 15.2 | 0.227 |
|  | 2 | 9 | 16 | 0.606 | 30.3 | 10.1 | 0.152 |
|  | 3 | 12 | 20 | 0.501 | 37.6 | 8.4 | 0.125 |
| **H1047L** | 1 | 4 | 9 | 0.698 | 17.5 | 11.6 | 0.175 |
|  | 2 | 5 | 11 | 0.402 | 20.1 | 6.7 | 0.101 |
|  | 3 | 6 | 12 | 0.303 | 22.7 | 5.1 | 0.076 |
| **H1047R** | 1 | 5 | 11 | 0.804 | 20.1 | 13.4 | 0.201 |
|  | 2 | 7 | 13 | 0.505 | 25.3 | 8.4 | 0.126 |
|  | 3 | 9 | 16 | 0.404 | 30.3 | 6.7 | 0.101 |

**Supplementary Table S8.** **LOB_95%_ and theoretical LOD_95%_ values for the PIK3CA assays.** The limit of blank (LOB_95%_), defined as the maximum number of false-positive droplets expected in a chamber with a probability of 95% (i.e. for α risk equal to 5%) in a sample containing no target sequence, and the theoretical limit of detection (LOD_95%_), defined as the minimum concentration that can be said to be non-zero and statistically higher than the limit of blank with a probability of 95%, were determined for the PIK3CA assays following the instructions provided by Stilla Technologies. First, we counted the number of false positive droplets obtained for each detection when testing 30 replicates of WT-only samples (gDNA from healthy donors), with theoretical concentrations (based on the Qubit quantifications) of at least 10,000 copies/PCR. The corrected means were then calculated using the following equation: $\mu_{\mathrm{corr}}=\mu+1.645 \sigma\sqrt{\text{N}}$, where $\mu$ is the mean, $\sigma$ the standard deviation of false positive events and $N$ the number of experiments performed. The LOB_95%_ were determined by fitting the $\mu_{\mathrm{corr}}$ on Normal Law approximation and Chernoff’s inequality and the LOD_95%_ were calculated, with a similar approach as in Milbury et al.^10^, using the following equation: $\mathrm{LOD}_{\mathrm{droplets}}(95\%)=\lceil-N ln(1-p_{0})\rceil$, where $N$ is the total number of droplets that are generated on average in a chamber and $p_{0}$ is the solution of the following equation: $p_{0}= \frac{2b + z^{2} + z\sqrt{z^{2} + 4b (1-\frac{b}{N})}}{2N (1 + \frac{z^{2}}{N})}$, where $b$ is the LOB_95%_ and $z$ is the ‘one-tail’ quantile at 95% (equal to 1.645). The LOD_95%_ is either expressed in droplets or in copies/µl of PCR Mix, the latter can be converted into copies/PCR (for 25 µl of PCR Mix per replicate) as well as in copies/ml of plasma (for 15 µl of input DNA, 50 µl of elution volume, and 5 ml of plasma) or in MAF (%) (for the maximum concentration of 10,000 copies of total cfDNA per replicate). Although these values are theoretical as plasma volumes and cfDNA concentrations vary for each patient.

| **PIK3CA Assay n°1** | | | | | | | | | | | | | | | | | | | | | | | |  |
| --- | --- | --- | --- | --- | --- | --- | --- | --- | --- | --- | --- | --- | --- | --- | --- | --- | --- | --- | --- | --- | --- | --- | --- | --- |
| **EXPECTED CONCENTRA-TION**  **(copies/PCR)** | **MEASURED CONCENTRATION (copies/PCR)** | | | | | | | | | | | | | | | | | | | | | | |  |
|  | **WT** | | | | | | | **N345K-C420R-H1047L/R** | | | | | | | | **542/6_MUT** | | | | | | | |  |
|  | **MEAN** | | **SD** | | | **CV (%)** | | **MEAN** | | | **SD** | | | **CV (%)** | | **MEAN** | | | **SD** | | | **CV (%)** | |  |
| **10,000** | 9,678.2 | | 321.1 | | | 3.3 | | 4,333.8 | | | 288.2 | | | 6.7 | | 9,026.0 | | | 228.6 | | | 2.5 | |  |
| **5,000** | 4,882.9 | | 150.3 | | | 3.1 | | 2,764.1 | | | 115.6 | | | 4.2 | | 5,228.0 | | | 143.7 | | | 2.7 | |  |
| **50** | 49.3 | | 10.4 | | | 21.2 | | 28.8 | | | 11.9 | | | 41.3 | | 52.1 | | | 6.2 | | | 11.8 | |  |
| **PIK3CA Assay n°2** | | | | | | | | | | | | | | | | | | | | | | | |  |
| **EXPECTED CONCENTRA-TION (copies/PCR)** | **MEASURED CONCENTRATION (copies/PCR)** | | | | | | | | | | | | | | | | | | | | | | | |
|  | **WT** | | | | **E542K** | | | | | **E545K** | | | | | **H1047L** | | | | | **H1047R** | | | | |
|  | **MEAN** | **SD** | | **CV (%)** | **MEAN** | | **SD** | | **CV (%)** | **MEAN** | | **SD** | **CV (%)** | | **MEAN** | | **SD** | **CV (%)** | | **MEAN** | **SD** | | **CV (%)** | |
| **10,000** | 9,111.4 | 456.1 | | 5.0 | 9,385.1 | | 187.4 | | 2.0 | 18,599.1 | | 594.8 | 3.2 | | 8,214.6 | | 394.4 | 4.8 | | 13,483.7 | 564.6 | | 4.2 | |
| **5,000** | 4,491.8 | 173.1 | | 3.9 | 5,879.5 | | 153.0 | | 2.6 | 8,795.6 | | 281.9 | 3.2 | | 4,962.3 | | 200.6 | 4.0 | | 6,755.0 | 222.8 | | 3.3 | |
| **50** | 46.5 | 12.4 | | 26.7 | 56.2 | | 9.5 | | 16.8 | 87.6 | | 15.1 | 17.2 | | 52.7 | | 10.3 | 19.6 | | 61.4 | 14.7 | | 23.9 | |

**Supplementary Table S9.** **Results obtained during the repeatability study for the PIK3CA assays.** Three DNA mixes were prepared for each multiplex assay by serial dilutions of MUT gBlocks and WT gDNA in order to obtain mixes with theoretical concentrations of 10,000, 5,000, and 50 copies/PCR for each detection. For the PIK3CA Assay n°1, a mix containing the four corresponding MUT gBlocks of the N345K-C420R-H1047L/R detection and the eight most frequent mutations identified on codons 542 to 546 (E542K, E545A/G/K/Q, Q546K/P/R) was prepared in order to reach the same theoretical concentrations considering the total amounts of MUT DNA per detection. Each DNA mix was tested in eight replicates during the same PCR experiments in order to study the repeatability of the measures; this was done by calculating the coefficient of variation (CV) using the following equation: $CV (\%)=\frac{\sigma}{\mu}\times100$, where σ is the standard deviation (SD) and µ the mean of the replicate results. Please note that due to their short length (166 bp), the mass represented by one copy of a mutated gBlock is extremely low (1.7.10^-10^ ng). Therefore, in order to reach the concentrations required to prepare these DNA mixes, each mutated gBlock stock solution must be diluted 1:1,000,000 via a cascade dilution strategy, as stock solutions are concentrated to 10 ng/µl, which is 5.9.10^10^ copies/µl. It is therefore not abnormal to observe marked differences between the ‘expected values’ and the ‘measured values’.

| **PIK3CA Assay n°1** | | | |
| --- | --- | --- | --- |
| **MUTATION** | **EXPECTED MAF (%)** | **MEASURED MAF MEAN (%)** | **ACCURACY (%)** |
| **E545K** | 8.60 | 7.70 | 90 |
| **H1047R** | 16.93 | 13.68 | 81 |
| **PIK3CA Assay n°2** | | | |
| **MUTATION** | **EXPECTED MAF (%)** | **MEASURED MAF MEAN (%)** | **ACCURACY (%)** |
| **E545K** | 8.60 | 8.25 | 96 |
| **H1047R** | 16.93 | 15.76 | 93 |

**Supplementary Table S10.** **Results obtained during the accuracy study for the PIK3CA assays.** The accuracy of the results obtained with the PIK3CA assays were evaluated using the Quantitative Multiplex gDNA Reference Standard kit (Horizon Discovery) by testing the mix in four replicates and using the following equation: $\mathrm{ACCURACY}\left( \% \right)= \frac{MEASURED MAF MEAN (\%)}{EXPECTED MAF (\%)} \times100$.

| **PATIENT** | **MUTATION (AA (MAF(%))** | | |
| --- | --- | --- | --- |
|  | **PIK3CA assays**  **(cfDNA)** | **Bio-Rad dPCR assays**  **(cfDNA)** | **SAFIR02 NGS results**  **(Tumor gDNA)** |
| **A** | E542K (2.00%) | E542K (2.57%) | NA |
| **B** | E542K (28.90%) | E542K (34.60%) | NA |
| **C** | E545K (2.12%) | E545K (2.38%) | NA |
| **D** | E545K (4.06%) | E545K (4.63%) | NA |
| **E** | E545K (5.06%) | E545K (6.29%) | NA |
| **F** | E545K (21.05%) | E545K (22.70%) | NA |
| **G** | H1047R (5.71%) | H1047R (5.67%) | NA |
| **H** | - | - | NA |
| **I** | - | - | NA |
| **J** | - | - | NA |
| **K** | - | - | NA |
| **L** | - | - | NA |
| **#20** | Q546K (2.06%) | NA | Q546K |
| **#21** | H1047R (37.57%)  E545K (0.17%) | NA | H1047R |
| **#56** | H1047R (6.18%) | NA | H1047R |
| **#131** | C420R (6.50%) | NA | C420R |

**Supplementary Table S11. Analytical specificity validation of the PIK3CA assays using commercial dPCR assays (Bio-Rad) or in comparison to the next-generation sequencing (NGS) results of the SAFIR02 study.** The results obtained with the PIK3CA assays were compared to those obtained using the ‘ddPCR™ Mutation Assay: PIK3CA, Human, Homo sapiens’ from Bio-Rad for p.E542K (dHsaMDV2010073), p.E545K (dHsaMDV2010075) and p.H1047R (dHsaMDV2010077) on the cfDNA samples of a small subset of 12 patients (patients ‘A’ to ‘L’, not included in the present study). We obtained a 100% concordance with the same mutations (E542K, E545K and H1047R) found for the 7 mutated cfDNA samples (patients ‘A’ to ‘G’) and no mutation found for the 5 other samples (patients ‘H’ to ‘L’). According to the informations provided in the validation reports of these commercial assays, they have a detection sensitivity of up to ‘~0.1%’, which is pretty similar to the values of sensitivity that we obtained for our assays. Moreover, the accuracies of the MAF (%) obtained for the mutated samples were very consistent. In addition, the PIK3CA assays results obtained on the cfDNA samples of a small subset of 4 patients (patients #20, #21, #56 and #131 of the present study (Supplementary Dataset)) were compared to the NGS results of the SAFIR02 study performed on tumor gDNA samples and the presence of the same mutations could be confirmed for all of these patients, with the exception of a low frequency E545K mutation (MAF = 0.17%) found with the PIK3CA assays in plasma that could not be revealed by NGS in the tumor sample.

*(-: negative result (no mutation found); AA: amino acid; MAF: mutant allelic frequency; NA: not applicable).*

|  | **COSMIC Database** | | **Results obtained on our series of plasma samples** | |
| --- | --- | --- | --- | --- |
| **MUTATION**  **(AA)** | **NUMBER OF MUTATIONS** | **FREQUENCY** | **NUMBER OF MUTATIONS** | **FREQUENCY** |
| H1047R | 1560 | 48.45% | 27 | 36.49% |
| E545K | 700 | 21.74% | 25 | 33.78% |
| E542K | 420 | 13.04% | 7 | 9.46% |
| H1047L | 199 | 6.18% | 6 | 8.11% |
| C420R | 53 | 1.65% | 3 | 4.05% |
| N345K | 111 | 3.45% | 2 | 2.70% |
| Q546K | 30 | 0.93% | 2 | 2.70% |
| Q546R | 22 | 0.68% | 1 | 1.35% |
| Q546P | 13 | 0.40% | 1 | 1.35% |
| E545A | 48 | 1.49% | 0 | 0.00% |
| E545G | 24 | 0.75% | 0 | 0.00% |
| E545Q | 13 | 0.40% | 0 | 0.00% |
| E545D | 6 | 0.19% | 0 | 0.00% |
| Q546E | 6 | 0.19% | 0 | 0.00% |
| Q546H | 4 | 0.12% | 0 | 0.00% |
| E542Q | 3 | 0.09% | 0 | 0.00% |
| E542V | 3 | 0.09% | 0 | 0.00% |
| E542A | 2 | 0.06% | 0 | 0.00% |
| Q546L | 2 | 0.06% | 0 | 0.00% |
| E542G | 1 | 0.03% | 0 | 0.00% |
| E542R | 0 | 0.00% | 0 | 0.00% |

**Supplementary Table S12. Comparison of mutation frequencies observed in the COSMIC database and in our series of plasma samples.** PIK3CA mutations are in order of decreasing frequency. The calculation takes into account only the mutations detected and characterized by our multiplex assays.
